# Nanotechnology in oncology: advances in biosynthesis, drug delivery, and theranostics

**DOI:** 10.1007/s12672-025-02664-3

**Published:** 2025-06-21

**Authors:** Mohamed M. Ammar, Rania Ali, Naira Ali Abd Elaziz, Heba Habib, Fatima M. Abbas, Mohamed Taha Yassin, Khalid Maniah, Rewan Abdelaziz

**Affiliations:** 1 Microbiology and Biochemistry Program, Faculty of Science, Benha University-Obour Campus, Benha, 13518 Egypt; 2 Department of Botany and Microbiology, Faculty of Science, Zagazig University, Zagazig, 44511 Egypt; 3 Department of Chemistry and Zoology, Faculty of Science, Cairo University, Giza, Egypt; 4 Chemistry Department, Faculty of Science, Suez Canal University, Ismailia , Egypt; 5 Biophysics Department, Cairo University, Giza, Egypt; 6 Department of Botany and Microbiology, College of Science, King Saud University, P.O. Box 22452, 11495 Riyadh, Saudi Arabia; 7 Department of Biology, King Khalid Military Academy, Riyadh, Saudi Arabia; 8 Department of Microbiology, Faculty of Science, Ain Shams University, Cairo, Egypt

## Abstract

Nanotechnology has revolutionized oncology by offering innovative solutions to overcome the limitations of conventional cancer therapies. This review explores the transformative potential of nanotechnology in cancer diagnosis, treatment, and drug delivery, emphasizing the development of sustainable nanocomposites derived from natural sources such as plants and microbes. These eco-friendly nanocomposites enhance therapeutic efficacy, minimize environmental impact, and align with green chemistry principles. Nanoparticles (NPs) enable targeted drug delivery through mechanisms like the enhanced permeability and retention (EPR) effect and active targeting, reducing systemic toxicity and improving treatment outcomes. They also facilitate gene therapy, photothermal and photodynamic therapies, and immune modulation, including the development of cancer vaccines and theranostic platforms. Despite their promise, challenges such as nanoparticle toxicity, immune clearance, and long-term biocompatibility persist. Advances in biodegradable and stimuli-responsive NPs aim to address these issues, ensuring safer and more effective applications. The integration of nanotechnology with personalized medicine and combination therapies holds significant potential for improving cancer treatment efficacy and patient outcomes. However, further research is needed to optimize nanoparticle design, enhance tumor targeting, and ensure clinical translation. This review highlights the critical role of nanotechnology in advancing cancer therapy, underscoring its potential to redefine treatment paradigms while addressing current limitations and future prospects.

## Introduction

Particles with at least one dimension measuring between 1 and 100 nm are referred to as nanoparticles (NPs) [1, 2]. The size range of 1–100 nm is critical for NPs to exhibit their unique properties and functionalities. At this nanoscale, NPs possess special characteristics, including a high surface area-to-volume ratio and the ability to exhibit quantum effects, which distinguish them from bulk materials [3]. NPs can be composed of a wide variety of materials, such as metals, metal oxides, semiconductors, polymers, and biological components. They can also take on diverse morphologies, including spheres, cylinders, tubes, and other shapes, depending on their intended application. These versatile properties make nanoparticles highly valuable in fields such as medicine, electronics, energy, and environmental science, enabling innovative solutions to complex challenges. However, their unique characteristics also require careful consideration of factors like biocompatibility, toxicity, and stability to ensure safe and effective use[1].

Nanoparticles are utilized across a wide range of industries, with significant applications in biomedicine. They play a transformative role in drug delivery, biosensors, and bioimaging, offering innovative solutions to complex medical challenges [4]. In cancer therapy, NPs enhance drug cytotoxicity while minimizing off-target effects, improving treatment outcomes. They are also employed in the biodetection of pathogens and the detection of proteins that interact with DNA structures, enabling early diagnosis and monitoring of diseases.

In tissue engineering, NPs contribute to the development of advanced scaffolds for cell growth and regeneration. Additionally, NPs are used in tumor destruction through techniques like hyperthermia, where they generate heat to selectively kill cancer cells. They also facilitate the separation and purification of biological molecules and cells, enhancing research and diagnostic capabilities. In medical imaging, NPs serve as contrast agents for MRI, improving the clarity and precision of imaging techniques.

Beyond cancer, NPs have shown promise in treating inflammatory diseases. They can modulate immune responses by inhibiting monocyte production and egress in the bone marrow, regulating monocyte recruitment, depleting macrophages, and neutralizing proinflammatory cytokines and endotoxins [4], such as the inhibition of monocyte production and egress in the bone marrow, bone marrow accumulation, monocyte recruitment modulation, macrophage depletion, the inhibition of macrophage proliferation, and the neutralization of endotoxins and proinflammatory cytokines [5].

Natural compounds sourced from microbes and plants have emerged as powerful candidates in the development of anticancer therapeutics, owing to their remarkable bioactive properties and multifaceted mechanisms of action. These compounds, with their diverse chemical structures and potent biological activities, offer a rich reservoir for discovering novel agents capable of targeting cancer cells with high specificity and minimal adverse effects. Their natural origin and evolutionary refinement further underscore their potential as foundational elements in the design of next-generation anticancer therapies [5].

Recent advancements in synthetic biology have revolutionized the development of anticancer medications derived from plants by leveraging genetically modified microbial organisms, offering a cost-effective and scalable approach to drug production [6]. Notably, over 60% of currently marketed anticancer drugs originate from natural sources, with a growing number of plant-derived bioactive compounds progressing through clinical trials, highlighting their immense therapeutic potential [7]. The integration of natural substances with anticancer medications or polymeric carriers designed for targeted tumor delivery holds promise for enhancing both the safety and efficacy of cancer treatments. By combining the bioactive properties of natural compounds with advanced drug delivery systems, researchers can achieve precision targeting of tumor sites while minimizing off-target effects [7]. Plants, in particular, are increasingly recognized as reliable and sustainable sources of anticancer compounds, providing viable alternatives to conventional therapies. A comprehensive systematic study identified 199 anticancer plants, with significant focus on their applications in treating skin and breast cancers, underscoring their potential to address some of the most prevalent and challenging malignancies. These developments highlight the transformative role of natural products and synthetic biology in shaping the future of cancer therapeutics [8].

Currently, over thirty plant-derived medications are under investigation in clinical trials, demonstrating remarkable promise in combating a wide range of cancer types [9]. Alongside microbial-derived agents such as bleomycin and doxorubicin, plant-based compounds like vincristine, paclitaxel, and etoposide have already proven their efficacy in cancer treatment. These natural substances exert their anticancer effects through diverse mechanisms, including inducing apoptosis, disrupting signaling pathways, and inhibiting angiogenesis [10]. Furthermore, microbial amphiphiles, such as lipopeptides and glycolipids, have also shown significant anticancer potential. These compounds target critical cellular processes, such as blocking oncogenic signaling pathways, suppressing tumor angiogenesis, and triggering programmed cell death, thereby hindering cancer progression [11]. The multifaceted actions of these natural compounds, combined with their low toxicity profiles, make them invaluable assets in the development of safer and more effective cancer therapies. Their continued exploration and integration into clinical practice hold immense potential for advancing precision oncology and improving patient outcomes.

By functionalizing nanoparticles (NPs) with ligands or antibodies that specifically bind to receptors on cancer cells, therapeutic agents can be delivered directly to tumor sites while sparing healthy tissues, thereby minimizing off-target effects [6]. This targeted approach is further enhanced by the enhanced permeability and retention (EPR) effect, which causes NPs to accumulate preferentially in tumor tissues due to the leaky vasculature and impaired lymphatic drainage characteristic of tumors [12–14]. Moreover, nanoparticles enable sustained and controlled drug release, which can be precisely regulated in response to external stimuli such as pH, temperature, or light, offering unparalleled therapeutic precision and efficacy [15].

Among the various types of NPs, silver nanoparticles (AgNPs) have gained significant attention for their inherent antibacterial and anticancer properties. AgNPs exert their effects through multiple mechanisms, including the release of silver ions and the generation of reactive oxygen species (ROS) upon interaction with bacterial or cancer cells. These actions disrupt critical cellular processes, leading to cellular damage and death [16]. Due to their potent antimicrobial and anticancer capabilities, AgNPs are already widely used in antiseptic medical bandages and other therapeutic applications. This dual functionality underscores the versatility and potential of AgNPs in both infection control and cancer therapy, making them a valuable tool in modern medicine [17].

Several nanoparticle-based formulations have already received regulatory approval for clinical use, demonstrating the clinical viability and therapeutic potential of nanotechnology in medicine. Notable examples include albumin-bound paclitaxel (Abraxane) and liposomal doxorubicin (Doxil), which have become integral to modern cancer treatment regimens. These formulations leverage the unique properties of nanoparticles to enhance drug solubility, improve targeted delivery, and reduce systemic toxicity, thereby offering significant advantages over conventional therapies. The successful clinical application of these nanoparticle-based drugs underscores the transformative impact of nanotechnology in advancing precision medicine and improving patient outcomes [18]. Compared to traditional chemotherapy, nanoparticles offer unprecedented precision in drug delivery, significantly enhancing therapeutic outcomes while minimizing adverse effects. This targeted approach allows for the selective accumulation of therapeutic agents at tumor sites, sparing healthy tissues and reducing systemic toxicity. As a result, the application of nanoparticles in cancer therapy represents a groundbreaking innovation with the potential to redefine treatment paradigms. With ongoing advancements in research, these technologies are poised to revolutionize cancer treatment, offering safer, more effective, and personalized therapeutic solutions for patients worldwide.

Nanocomposites (NCs) represent a distinct class of nanomaterials where one or more phases with nanoscale dimensions—ranging from zero-dimensional (0D), one-dimensional (1D), to two-dimensional (2D) structures—are embedded within a ceramic, metallic, or polymeric matrix. These nanocomponents exhibit unique biological effects on cancer cells, contributing to their anticancer properties. The therapeutic potential of NCs is attributed to several key features, including their ability to induce apoptosis (programmed cell death) and their antioxidant properties, which collectively hinder cancer progression.

Among the various types of nanocomposites (NCs), zinc–sodium alginate–ethylene glycol–brucine (ZAP–Brucine) nanoparticles have demonstrated remarkable efficacy in targeting and overcoming cancer cells, particularly gallbladder cancer cells (GBCs). These nanoparticles leverage their unique composition and nanoscale properties to deliver potent anticancer effects, making them a promising candidate for targeted cancer therapy. The ability of ZAP–Brucine NPs to selectively act on cancer cells while minimizing damage to healthy tissues highlights their potential as an innovative therapeutic strategy in the cancer treatment [19]. The rare earth-iron mixed oxide/magnetic oxide/reduced graphene oxide (ErFeO_3_/Fe_3_O_4_/rGO) nanocomposite, also referred to as ErFeNP/FeNP/rGO, exhibits significant anticancer activity and is synthesized through an eco-friendly green synthesis approach using sucrose sugars. This nanocomposite leverages the synergistic properties of its components to deliver potent therapeutic effects against cancer cells.

A critical aspect of characterizing such nanocomposites (NCs) involves their detection and analysis, which is accomplished using a suite of advanced analytical techniques. These include:

- Transmission Electron Microscopy (TEM) and High-Resolution Transmission Electron Microscopy (HRTEM) for detailed imaging of nanoscale structures and morphology.
- Scanning Electron Microscopy (SEM) to examine surface topography and composition.
- X-Ray Diffraction (XRD) for crystallographic analysis and phase identification.
- Brunauer–Emmett–Teller (BET) and Barrett-Joyner-Halenda (BJH) analyses to determine surface area and pore size distribution.
- Vibrating Sample Magnetometry (VSM) to assess magnetic properties.

These techniques collectively provide comprehensive insights into the structural, morphological, and functional properties of nanocomposites, enabling researchers to optimize their design and performance for a wide range of biomedical and therapeutic applications. The integration of these advanced characterization methods ensures the development of highly effective and tailored nanomaterials for innovative cancer therapies [20].

## Background on nanocomposite types and their applications in anticancer and drug delivery

The concept of nanotechnology was first introduced by Richard Feynman in his groundbreaking 1959 lecture titled "There’s Plenty of Room at the Bottom," delivered at the California Institute of Technology (Caltech). In this lecture, Feynman envisioned the potential of manipulating matter at the atomic and molecular scale. Later, the term "nanotechnology" was formally coined by Professor Norio Taniguchi of Tokyo Science University in 1974, marking a pivotal moment in the field's development [21].

Green materials derived from microbes, plants, fruits, and other natural sources are increasingly utilized in the synthesis of metallic nanoparticles, offering a sustainable and eco-friendly approach. The use of biowaste materials further enhances this process, enabling the production of highly dynamic, biocompatible nanoparticles that contribute to environmental sustainability, waste recycling, and targeted cancer cell therapy. These nanoparticles also facilitate the delivery of large molecules for drug delivery applications. In recent years, nanotechnology has expanded its reach across diverse fields, including biology, chemistry, physics, medicine, and pharmacy, demonstrating its transformative potential in both scientific research and practical applications [22].

Metallic nanoparticles, first discovered by Michael Faraday in 1857, encompass a wide range of metal and metal oxide nanoparticles, including silver, gold, iron, zinc, copper, magnesium, and graphene. These nanoparticles have since become foundational materials in nanotechnology, offering unique properties and applications across various scientific and industrial fields [23]. Metal and metal oxide nanoparticles (M/MO-NPs) are among the most widely used types of nanoparticles due to their significant roles in antifungal and antibacterial activities, cancer treatment, drug delivery, and diagnostic applications. Their unique properties and versatility make them indispensable in advancing biomedical and therapeutic technologies [24].

Recently, multifunctional hydrogels derived from homo- and copolymers, such as tragacanth gum-based hydrogels (TGIAVE) and TGIAVE-Ag, have garnered significant attention for their applications in drug delivery, cancer treatment, and the inactivation of multidrug-resistant bacteria [25]. These nanocomposites represent a promising advancement in biomedical research. Similarly, magnesium oxide nanoparticles (MgO NPs), synthesized through green methods, are widely used in drug delivery and anticancer applications due to their high biocompatibility [26]. Biocompatibility, defined as the ability of a biomaterial to perform its intended function without causing toxic or harmful effects on biological systems, is a critical factor in the development of safe and effective therapeutic nanomaterials [27].

The application of nanocomposites and nanoparticles in drug delivery systems represents a rapidly growing market, with current costs estimated between 510 and 134 billion US dollars. This market is projected to expand at a compound annual growth rate (CAGR) of 6.9%, reaching an estimated value of 900 to 293 billion US dollars by 2025. This growth underscores the increasing reliance on nanotechnology for advanced drug delivery solutions and its transformative impact on the pharmaceutical and biomedical industries [2].

Currently, many researchers are exploring the use of natural compounds extracted from plants, microbes, and other biological sources for cancer treatment. These compounds are cost-effective, highly potent, biocompatible, biodegradable, and exhibit low toxicity, making them ideal candidates for therapeutic applications. Among these, various metal oxide nanoparticles, such as titanium oxide (TiNPs), zinc oxide (ZnNPs), and magnesium oxide (MgNPs) nanoparticles, have demonstrated significant anticancer effects in both in vitro and in vivo studies, highlighting their potential as effective and sustainable alternatives in oncology [13]. In this context, M/MO-NPs exhibit remarkable properties, including high cellular uptake efficiency, controlled toxicity, and non-immunogenic responses, making them ideal carriers for genes, proteins, peptides, and small molecules in drug delivery systems. Their versatility and biocompatibility enable precise and effective therapeutic delivery, positioning them as key tools in advancing targeted and personalized medicine [23]. Table 1 showed different types of nanomaterials and their potential biomedical applications.

**Table 1:** Different types of nanomaterials and their biomedical applications

No	Nanocomposite	Applications	References
1	AgNPs	As antifungal activity, antibacterial activity, cancer treatment, drug delivery and diagnosis	[28–30]
2	AuNPs	As antifungal activity, antibacterial activity, cancer treatment, drug delivery and diagnosis	[31–34]
3	FeNPs	As antifungal activity, antibacterial activity, cancer treatment, drug delivery and diagnosis	[35–37]
4	ZnONPs	As antifungal activity, antibacterial activity, cancer treatment, drug delivery and diagnosis	[38–40]
5	CuNPs	As antifungal activity, antibacterial activity, cancer treatment, drug delivery and diagnosis	[41]
6	MgNPs	As antifungal activity, antibacterial activity, cancer treatment, drug delivery and diagnosis	[42]
7	GoNPs	As antifungal activity, antibacterial activity, cancer treatment, drug delivery and diagnosis	[43]
8	TGIAVE-Ag	As drug delivery to cancer treatment	[3]
9	MgO NPs	As high biocompatibility in drug delivery and cancer treatment	[44]
10	TiO_2_	As metal oxide NPs used in anticancer effects in vitro and in vivo	[45]
11	ZAP- Brucine	As overcoming cancer cells especially, gallbladder cells(GBC)	[46]

### The need for sustainable nanocomposites for anticancer and drug delivery

Sustainable nanocomposites are used in drug delivery systems to increase the therapeutic efficacy of drugs without any side effects on patients and enhance patient compatibility [47]. Tragacanth ® gum-based hydrogel (TGIAVE) ® hydrogels at pH values of 1.2 and 7.4 and temperatures of 25 and 37 °Care anticancer drugs. TGIAVE-Ag ® is a nanocomposite used for the inhibition of multidrug-resistant (MDR) bacteria [25].

Sustainable nanocomposites for anticancer use are MgO NPs, which have chemotherapeutic effects on fast diagnosis and the discovery of various types of cancer cells [48]. In recent studies, nanobiotechnology research has attracted great interest for various biomedical applications, such as the ability to inhibit cancerous cell growth, therapeutic processes such as drug delivery, and biomolecule sensors [49].

### The potential of using plants and microbes to create sustainable nanocomposites

Microorganisms, including bacteria, fungi, and algae, serve as promising biological sources for the sustainable synthesis of metal and metal oxide nanoparticles (M/MO-NPs). While microbes offer significant potential for nanoparticle production, their utilization is often considered less extensive compared to plant and fruit extracts, which are more widely employed in the creation of nanocomposites due to their accessibility, scalability, and rich bioactive compounds. This preference highlights the versatility of plant-based systems in green synthesis, despite the considerable potential of microbial systems in nanoparticle fabrication [50]. Biowaste materials, including fruit and vegetable peels, seeds, chicken eggshells, poultry feathers, tea waste, sugarcane bagasse, phytoplankton, human hair, agricultural weeds, cow dung, and urine, have emerged as valuable resources for the production of nanoparticles. These materials are rich in lignin, proteins, pectin, hemicelluloses, cellulose, and other biodegradable polysaccharides, as well as bioactive compounds such as flavonoids, carotenoids, polyphenols, and essential oils. However, their utilization requires careful consideration due to challenges such as potential toxic effects, scalability issues, and environmental sustainability concerns. Despite these challenges, biowastes represent a promising and eco-friendly alternative for nanoparticle synthesis, aligning with the principles of green chemistry and sustainable development [2].

Additionally, plant-based materials, such as extracts from *Terminalia bellirica* seeds, are utilized in the creation of nanocomposites (NCs), including the development of TGIAVE-Ag® through green synthesis. TGIAVE hydrogels® , which exhibit stability at pH values of 1.2 and 7.4 and temperatures of 25 and 37 °C, have demonstrated significant potential as anticancer agents. Furthermore, TGIAVE-Ag nanocomposites are effective in inhibiting multidrug-resistant (MDR) bacteria, showcasing their dual therapeutic capabilities. These hydrogels, composed of monomers and polymers derived from natural resources such as cellulosics, gums, sugars, fibers, and oils, are not only biodegradable but also environmentally sustainable, making them a promising candidate for advanced biomedical applications [24].

**Fig. 1:**
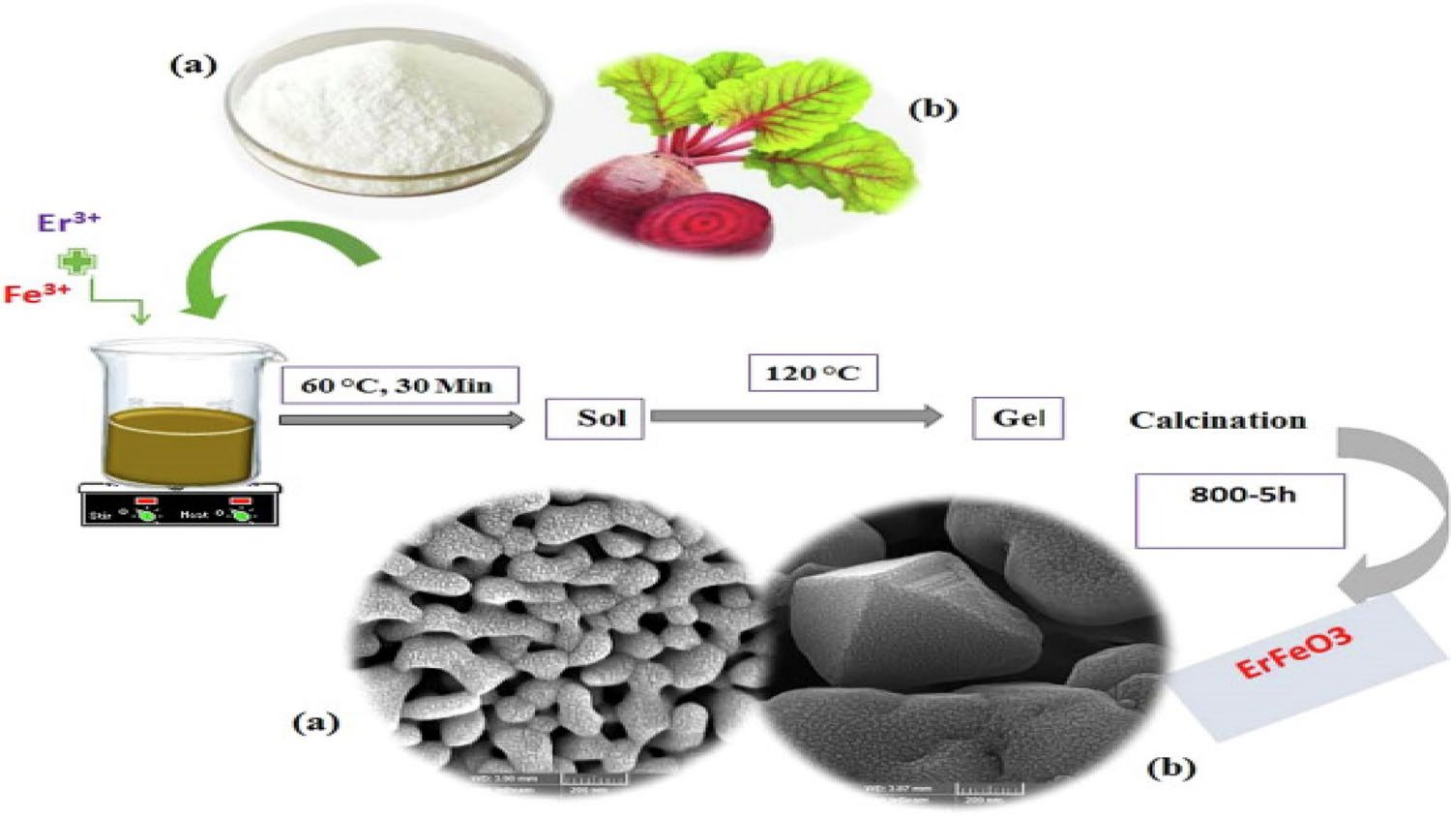
Erbium iron oxide nanocrystalline powders synthesized via the sol‒gel-combustion method using sugar and sugar beet as combustion agents [51]

Erbium iron oxide nanoparticles (ErFeO3 NPs) exhibit a unique structural configuration and remarkable chemical stability, making them highly suitable for applications in photocatalysis and magnetism. These nanoparticles are synthesized through an eco-friendly combustion process utilizing sugar beet plants, highlighting their sustainable production method and potential for integration into green technologies (figure 1) [51]. Hydrogels are also employed as nanocomposites, incorporating monomers and polymers derived from natural resources such as plant-based materials, including cellulosics, gums, sugars, fibers, and oils. This composition not only enhances their biocompatibility but also ensures their biodegradability, making them environmentally sustainable and suitable for a wide range of biomedical and industrial applications [52].

Surface functionalization processes in bacterial cellulose nanofibrils (BCs) are employed to improve the adhesion between BC and hydrophobic polylactide (PLLA), facilitating the development of environmentally friendly green nanocomposites. This functionalization is achieved using various organic acids, such as acetic, hexanoic, and dodecanoic acids, which modify the surface properties of BC, enhancing its compatibility with PLLA and enabling the creation of sustainable, high-performance materials [53].

The contact angle of the polymer droplets on BC is determined via the direct wetting method [31, 32]. The utilization of microbes for the synthesis of nanocomposites is widely regarded as an eco-friendly and sustainable technology. Nanocomposite production can occur through either intracellular or extracellular mechanisms. Intracellular synthesis involves the use of bacterial biomass to form nanocomposites within the microbial cells, while the extracellular approach, also referred to as the post-culture method, leverages the supernatant—a solution rich in biologically active compounds such as enzymes and metabolites—after the removal of microbial cells. Both methods offer unique advantages, contributing to the development of green and efficient nanocomposite fabrication processes.

The synthesis of nanocomposites using microbes results in nanoparticles with diverse shapes and sizes, influenced by factors such as microbial species, pH, temperature, and substrate concentration. The production mechanisms often involve electrostatic interactions between the negatively charged cell walls of microbes and positively charged metal ions, followed by enzymatic reduction of these ions into nanoparticles. For instance, *Rhodobacter capsulatus* secretes NADH-dependent enzymes that facilitate the reduction of gold ions to gold nanoparticles [54].

Fungi, including species such as *Verticillium luteoalbum*, *Colletotrichum *sp., *Fusarium oxysporum*, *Trichothecium* sp., *Aspergillus oryzae*, *Alternaria alternata*, and *Trichoderma viride*, are particularly significant due to their ability to secrete large quantities of proteins, enhancing nanoparticle productivity. These fungi are cultivated on a large scale using solid substrate fermentation, yielding substantial biomass for nanocomposite production. Bacteria, fungi, and yeast are favored for their rapid growth, ease of cultivation, and adaptability to controlled environmental conditions, such as high temperature, pressure, and pH. Bacterial-mediated reduction pathways minimize the need for toxic chemicals in nanoparticle synthesis. For example, *Aquaspirillum magnetotacticum* and *Magnetospirillum magnetotacticum* produce iron oxide nanoparticles, while *Shewanella oneidensis* and *Desulfosporosinus* sp. synthesize uranium dioxide nanoparticles [55].

**Fig. 2:**
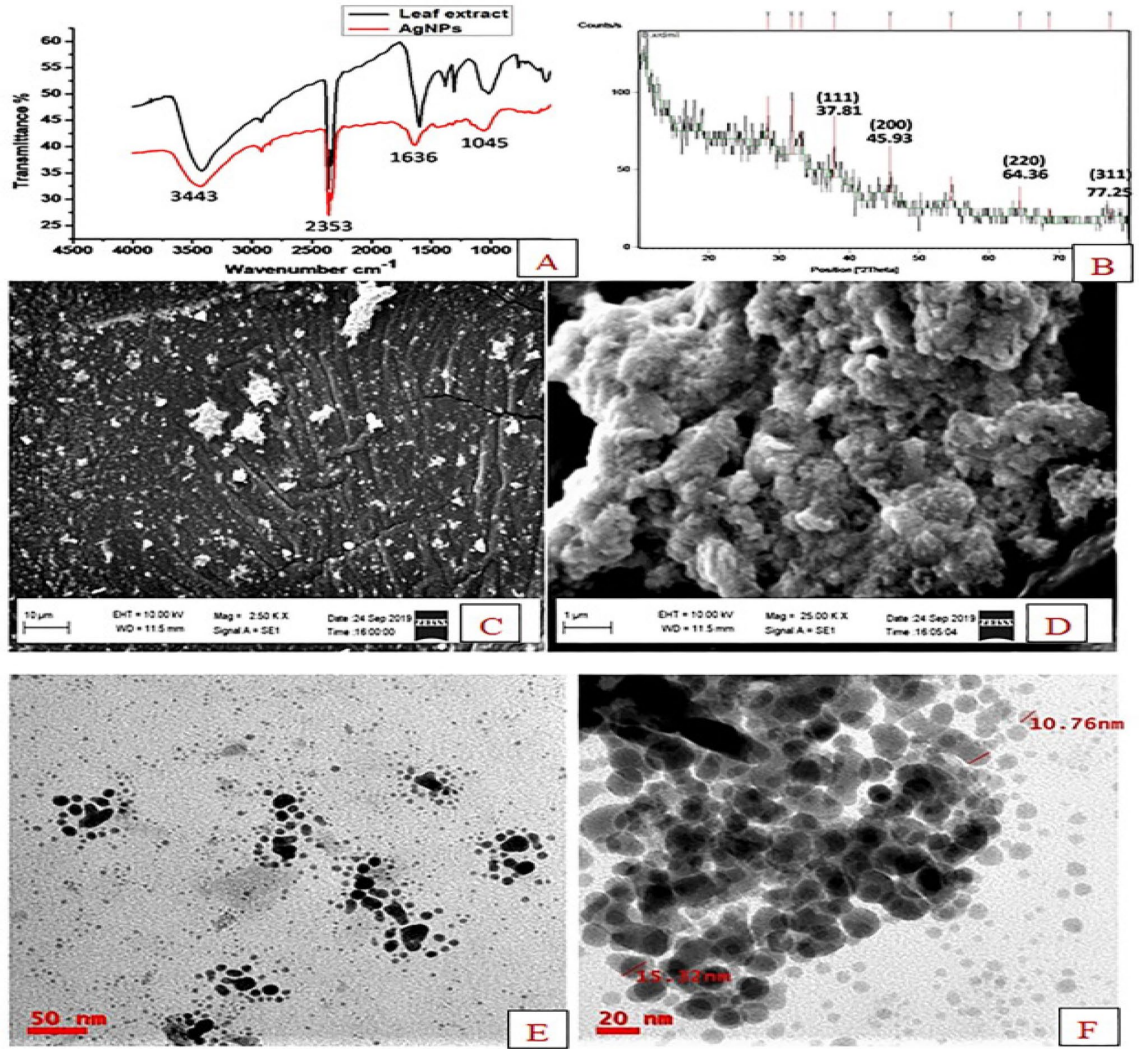
Characterization of Green-mediated synthesized silver nanoparticles **a** Vibrational bands observed in the FTIR spectra of the *Cyphostemma* plant extract and green-synthesized CA-AgNPs. **b** The 0.2θ values shown in the XRD pattern of CA-AgNPs. **c** Scanning electron micrograph of green-mediated CA-AgNPs at 10 µm resolution. **d** Scanning electron micrograph of green-mediated CA-AgNfigurePs at 01 µm resolution. **e**. 50 nm magnification transmission electron microscopy images of green, synthetic CA-AgNPs. **f** 20 nm magnification transmission electron microscopy images of green, synthetic CA-AgNPs. AgNPs synthesized from *Cyphostemma auriculatum* extract act as inhibiting agents for the proliferation of cancer cells [8]

**Fig. 3:**
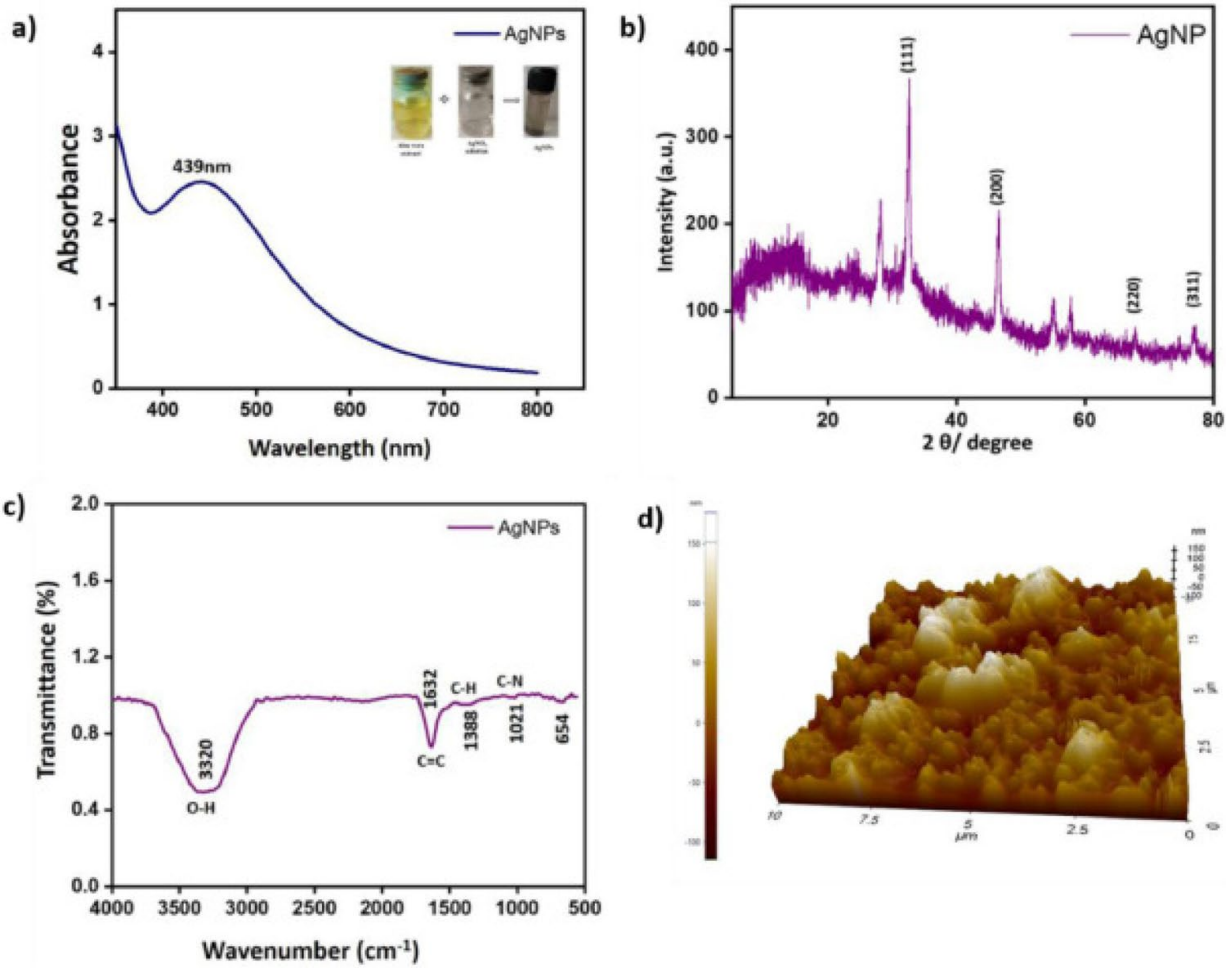
**a** UV–visible spectrum of the AgNPs synthesized from *Aloe vera* leaf extract at 439 nm, **b** XRD spectrum of the AgNPs, **c** ATR-IR spectra of the AgNPs and **d** AFM image of the AgNPs [10]

In recent years, silver nanoparticles (AgNPs) have garnered significant attention for their antioxidant and anticancer properties, coupled with minimal side effects. AgNPs synthesized from *Cyphostemma auriculatum* extracts, referred to as CA-AgNPs, demonstrate notable antitumor and antioxidant activities. These biogenic CA-AgNPs exhibit UV–Vis absorption peaks in the range of 390–450 nm. Optimal synthesis conditions, achieved using 8% of a 1 mM AgNO_3_ solution combined with 2% *Cyphostemma auriculatum* Roxb extract, produce nanoparticles with the highest intensity and sharpest spectral peaks. This highlights the potential of plant-mediated green synthesis for developing biocompatible and functionally advanced nanomaterials with therapeutic applications [56]. Moreover, other investigation revealed that AgNPs synthesized from *Cyphostemma auriculatum* extract, demonstrate potent anticancer activity by effectively inhibiting the proliferation of cancer cells, as evidenced by their structural and functional characterization (Fig. 2) [8]. In addition, AgNPs synthesized from *Aloe vera* leaf extract revealed potential antioxidant and antiproliferative activities against MCF-7 breast cancer cell line (Fig. 3).

### Advantages of using plants and microbes for nanocomposite synthesis

The use of plant-based materials, such as orange fruit peel extracts, for nanocomposite synthesis offers significant advantages, including the production of simple and eco-friendly zinc oxide nanoparticles (NPs), as demonstrated previously [57]. Additionally, hydrogels like TGIAVE-Ag, derived from *Terminalia bellirica* seeds and optimized at specific pH values and temperatures, exhibit controlled drug release properties, making them highly effective for targeted cancer treatment in drug delivery applications. These plant-mediated synthesis methods not only enhance sustainability but also provide a versatile platform for developing advanced therapeutic nanomaterials [3].

There are several plant-derived metal-based nanomaterials used as anticancer agents, such as gold (Au) and silver (Ag) NPs [58]. The incorporation of polyethylene glycol (PEG) with betulinic acid (BA) in drug delivery systems significantly enhances drug solubility and improves specificity for targeting cancer cells [59]. ZAP-Brucine nanoparticles (NPs), a type of nanocomposite, demonstrate remarkable potential in overcoming cancer cells by inducing cytotoxicity while sparing normal cells, highlighting their selective therapeutic efficacy and safety in anticancer applications [46]. In the present study, extracts from Pudeena plant leaves are utilized in the green combustion synthesis of barium oxide (BaO) NPs, ferric oxide (Fe_2_O_3_) NPs, and nickel oxide (NiO) NP nanocomposites, which are subsequently calcined at 500 °C for 3 hours. The structural and compositional properties of the BaO NP-Fe2O3 NP-NiO NP nanocomposites were analyzed and compared using powder X-ray diffraction (PXRD), providing insights into their crystallinity and intensity profiles, which are critical for optimizing their functional applications [9].

**Fig. 4:**
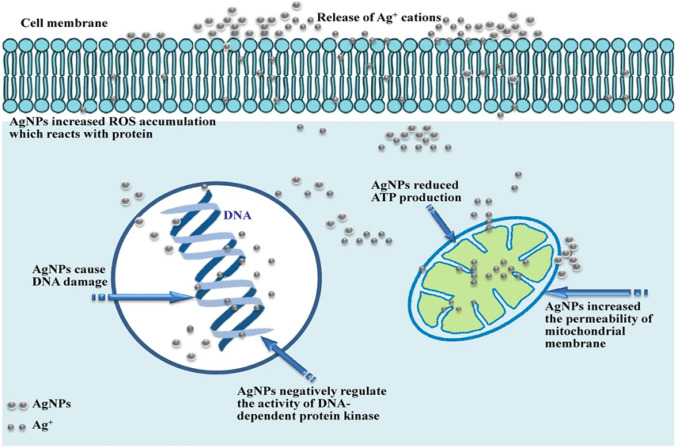
Mode of action of AgNPs against MCF7 cancer cell line [61]

Silver nanoparticles (AgNPs) synthesized from *Aloe vera* leaf extracts have demonstrated significant efficacy in eliminating breast cancer (MCF-7) cells. Figure 4 revealed demonstrated the possible mode of action of AgNPs against MCF7 cancer cell line. Silver nanoparticles (AgNPs) exert cytotoxic effects through multiple mechanisms. They release Ag⁺ ions that disrupt cell membrane integrity and increase permeability while inducing oxidative stress by generating reactive oxygen species (ROS) which damage proteins, lipids and mitochondria, leading to ATP depletion and apoptosis. Additionally, AgNPs directly interact with DNA, causing strand breaks and mutations while also inhibiting DNA dependent protein kinase (DNA PK), impairing DNA repair and exacerbating genomic instability. These combined actions including membrane disruption, oxidative stress, DNA damage and metabolic suppression result in cell death, making AgNPs effective antimicrobial and anticancer agents, though their therapeutic use requires careful toxicity management. These AgNPs offer numerous advantages in cancer treatment, including simplicity of synthesis, high efficiency, minimal side effects, biocompatibility, cost-effectiveness, and eco-friendliness. Their potent antioxidant properties enable the neutralization of free radicals and reactive oxygen species (ROS), protecting healthy cells while inducing cancer cell death, making them a promising therapeutic agent in oncology [60].

### Overview of anticancer properties

Cancer remains one of the leading causes of death globally, with its progression and prevention strongly influenced by dietary factors. Previous studies have highlighted the chemopreventive potential of phenolic compounds found in vegetables and fruits, which play a significant role in mitigating cancer risk. Despite advances in cancer surgery and therapeutic drugs, their efficacy remains limited in many cases, underscoring the need for alternative strategies [62].

Historically, significant efforts have been directed toward identifying alternative pathways to traditional chemoprevention, focusing on key signal transduction pathways such as nuclear factor-κB (NF-κB), activator protein-1 (AP-1), and mitogen-activated protein kinases (MAPKs). Polyphenols, known for their ability to induce apoptosis in cancer cells by modulating these pathways, have emerged as promising agents. However, the therapeutic use of dietary polyphenols is not without challenges, as they can trigger inflammatory responses and alter gene expression, potentially leading to adverse effects in patients. This highlights the need for careful optimization of polyphenol-based therapies to maximize their anticancer benefits while minimizing unintended consequences [63].

Several conventional treatment options, including radiation, chemotherapy, and surgery, are widely used in anticancer therapy; however, these methods often come with significant side effects. In contrast, self-assembling molecular drugs represent a promising advancement in cancer treatment, offering a unique combination of molecular chemotherapy and targeted tumor delivery through drug-nanoparticle conjugates. This innovative approach enhances therapeutic precision while minimizing adverse effects, paving the way for more effective and patient-friendly anticancer strategies [64].

**Fig. 5:**
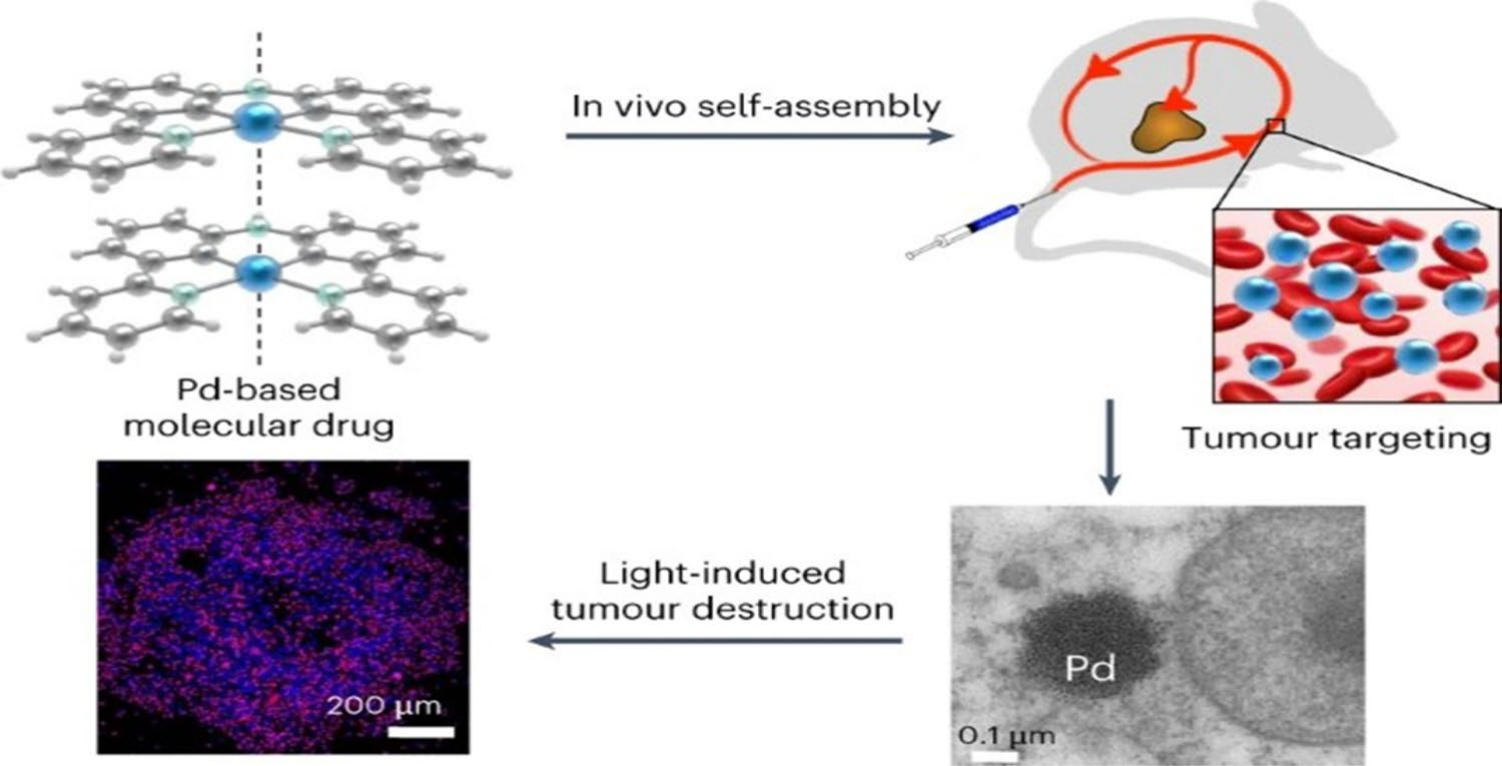
Self-assembled nanoparticles driven by Pd···Pd metallophilic interactions enable high tumor accumulation and type I photodynamic action under light activation, offering enhanced stability and therapeutic efficacy compared to conventional PDT [65]

A recent study reported a self-assembling palladium(II) complex (PdL) that formed stable nanoparticles in vivo via Pd···Pd metallophilic interactions, enabling effective tumor targeting and light-activated therapy. (Fig. 5). Metallophilic self-assembly of palladium complexes forms tumor-targeting nanoparticles, enabling light-activated photodynamic therapy with high drug-loading and minimal off-target toxicity [65].

### Mechanisms of action of some metal nanocomposites

Brucine nanoparticles (NPs) have been shown to modulate the Wnt/β-catenin signaling cascade, underscoring their anticancer potential. Specifically, ZAP-Brucine NPs exhibit significant anticancer effects on gallbladder cancer (GBC) NOZ cell lines. Experimental results indicate that these NPs effectively downregulate the PI3K/Akt/mTOR pathway in NOZ cells, further supporting their role as a promising therapeutic agent for targeting cancer signaling pathways and inhibiting tumor progression [46]. Ferric oxide (FeO_3_) nanoparticles (NPs) are recognized for their application in hyperthermal therapy, leveraging specific mechanisms to target cancer cells. When subjected to an alternating magnetic field (AMF), the dipole relaxation of FeO_3_ NPs generates significant heat, which selectively destroys cancer cells. While normal tissues may also be affected, the impact is minimal, ensuring a favorable therapeutic ratio. This targeted approach highlights the potential of FeO_3_ NPs as an effective and precise tool in cancer treatment [66]. An MTT cytotoxicity assay was employed to evaluate the response of breast cancer (MCF-7) cells following treatment with silver nanoparticles (AgNPs). The anticancer effects of AgNPs are mediated through multiple mechanisms, including the generation of free radicals, activation of key enzymes such as Caspase-3, release of mitochondrial components from the outer membrane, and DNA degradation. These processes collectively induce cancer cell death via necrosis, apoptosis, and autophagy, demonstrating the multifaceted therapeutic potential of AgNPs in breast cancer treatment [67].

**Fig. 6:**
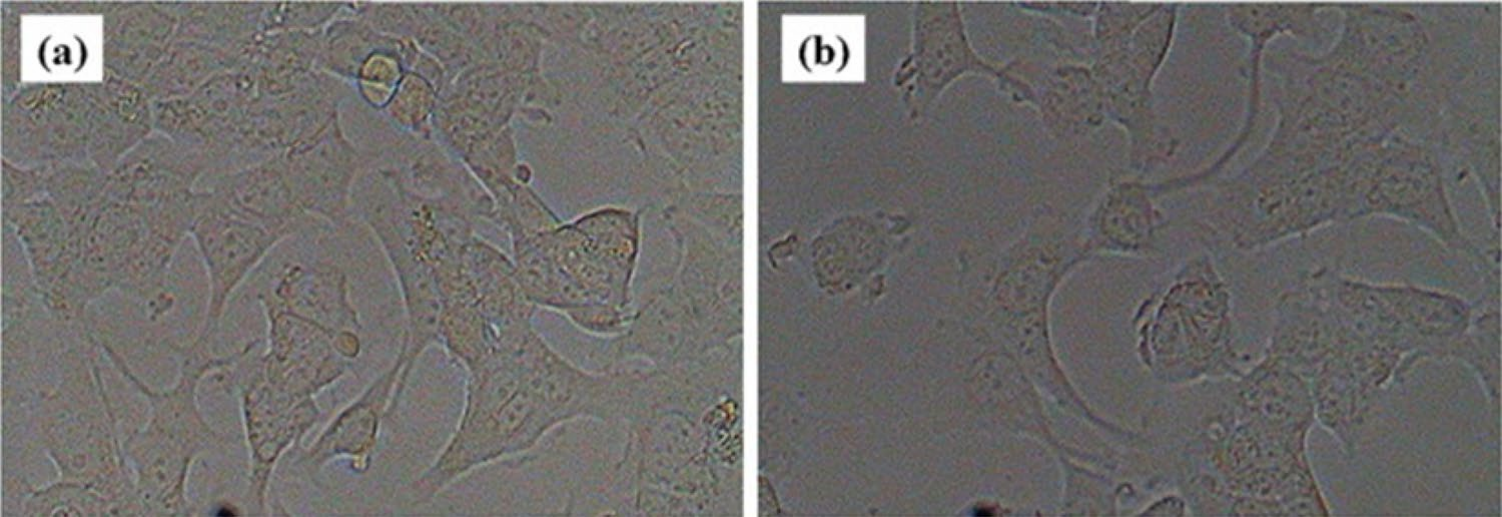
Anticancer activity of CA-AgNPs on MCF7 cells. **a** Control **b** Treatment shows the percent viability of silver nanoparticles (98, 88, 80, 72, 49, and 33%) at different concentrations (10, 20, 40, 60, 80, and 100 µg/mL) [8]

Additionally, silver nanoparticles (AgNPs) induce the degradation of proteins within cancer cells, leading to their complete elimination. In the control process, MCF-7 cells were treated without AgNPs, while the treatment process required the addition of nanoparticles, both of which significantly impacted cancer cell viability. The data reveal an apoptosis-dependent pathway, where higher concentrations of CA-AgNPs correlated with increased apoptosis rates. The IC_50_ (inhibitory concentration at 50%) of AgNPs was determined to be 70.6 µg/mL. Furthermore, the percentage viability of cancer cells decreased progressively with increasing AgNP concentrations, showing viability rates of 98%, 88%, 80%, 72%, 49%, and 33% at concentrations of 10, 20, 40, 60, 80, and 100 µg/mL, respectively. These findings underscore the dose-dependent cytotoxic effects of AgNPs on cancer cells (Fig. 6).

### Types of sustainable nanocomposites with anticancer properties

**Fig. 7:**
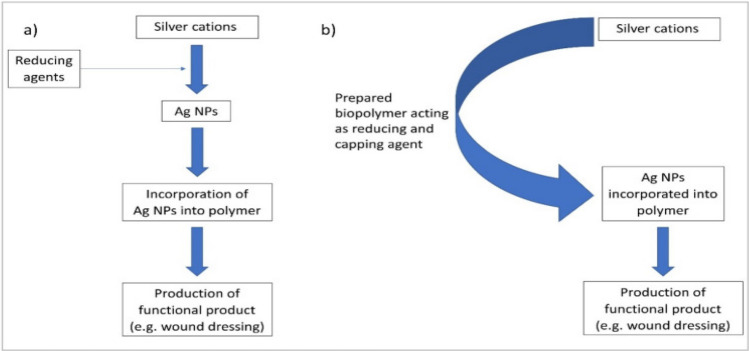
Examples of the production of Ag NPs followed by the capping of functional AgNPs with biopolymers [25]

Brucine nanoparticles (NPs) demonstrate potent anticancer activity against various cancer types, including colorectal cancer, by effectively inhibiting tumor cell growth [68]. Similarly, superparamagnetic iron oxide nanoparticles, such as ferric oxide (FeO_3_), are widely utilized in cancer therapy due to their excellent biocompatibility, low toxicity, and minimal side effects compared to conventional chemotherapy and radiotherapy. FeO_3_ NPs are particularly valuable in hyperthermal cancer therapy, where they generate localized heat to destroy cancer cells [69]. Additionally, hyaluronic acid-silver nanocomposites (HA-AgNPs) have emerged as promising biomedical agents for anticancer applications. These nanocomposites are synthesized by reducing silver cations and subsequently coating the resulting silver nanoparticles (Ag NPs) with biopolymers, enhancing their stability and therapeutic efficacy (Fig. 7) [25].

Silver nanoparticles (AgNPs) synthesized from *Aloe vera* leaf extracts have been effectively used to target and eliminate breast cancer (MCF-7) cells. These AgNPs exhibit significant anticancer activity at concentrations of 10 µg/mL or higher, with their efficacy increasing proportionally with concentration [10]. Similarly, platinum nanoparticles (PtNPs) have demonstrated potent activity against A431 and MCF-7 cancer cells [70]. The MTT assay further confirmed the cytotoxic effects of CA-AgNPs on MCF-7 cells, revealing that higher concentrations of CA-AgNPs induce the highest percentage of apoptotic cells, underscoring their potential as a powerful therapeutic agent in cancer treatment [56]. Table 2 highlights several polymer nanocomposites that have demonstrated antitumor activity. These composites typically combine polymers with functional nanoparticles, such as metallic or organic nanostructures, to enhance their therapeutic efficacy.

**Table 2:** The applications of certain polymer nanocomposites in cancer therapy

No	Nanocomposites	Applications	References
1	Brucine NPs	Overcoming hepatocellular carcinoma cells in vivo	[71]
2	FeO_3_ NPs	Cancer hyperthermal therapy	[72]
3	HA AgNPs	Anticancer and drug delivery activities	[25]
4	AgNPs	Antitumor agent of breast cancer (MCF-7)	[73]
5	Chitosan stabilized PtNPs	Overcoming MCF-7 tumor cells	[74]
6	CA-AgNPs	on MCF-7 cancerous cells	[75]

**Fig. 8:**
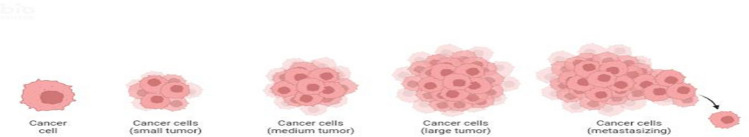
Growth of a tumor derived from a cancer cell

Over time, our understanding of cancer has evolved significantly, from the early observations of Hippocrates to the advanced scientific studies of today. Numerous factors, including nutrition, smoking, alcohol consumption, and environmental conditions, have been identified as contributors to cancer development. Since the FDA approved chemotherapy in the 1970s, treatment options have expanded to include hormone therapy, immunotherapy, radiation, surgery, and stem cell transplantation [76]. Despite these advancements, cancer remains a formidable global health challenge, with 19.3 million cases and 10 million deaths projected for 2020 [77]. Cancer development is a complex, multifactorial process involving genetic alterations triggered by radiation, toxins, ultraviolet light, and other stressors. These mutations lead to abnormal cell survival and proliferation, with cancer cells primarily relying on fermentation for energy, unlike normal cells that depend on respiration [78]. Cancer can be viewed as a form of reverse evolution, driven by mutation and selection [79]. Key players in cancer development include oncogenes, which promote uncontrolled cell division, and tumor suppressor genes, which typically inhibit cancer progression [80]. Additional mechanisms, such as the inhibition of apoptosis and angiogenesis, as well as the loss of cellular "social" behavior, further contribute to cancer growth. Cancer is categorized into four main types: carcinomas, sarcomas, leukemias, and lymphomas, each originating from different tissues—epithelial cells, connective tissues, blood-forming tissues, and the lymphatic system, respectively [81]. Figure 8 reveals the progressive development of cancer from a single abnormal cell to a metastatic stage. Initially, a single cancer cell undergoes uncontrolled division, forming a small tumor. As proliferation continues, the tumor enlarges, transitioning from a medium to a large mass with an increasingly dense cluster of cancer cells. In the final stage, metastasis occurs, where a cancerous cell detaches from the primary tumor and moves away, signifying its potential spread to other parts of the body. This illustration highlights the gradual progression of tumor growth and the critical stage of metastasis, which is a key factor in cancer severity and treatment challenges.

Lung cancer, for instance, is divided into small cell lung carcinoma (SCLC) and non-small cell lung carcinoma (NSCLC), each with distinct risk factors, genetic profiles, and treatment approaches [82]. Current therapeutic strategies include cryotherapy, photodynamic therapy, proton therapy, radiotherapy, chemotherapy, and surgery [83]. However, cancer stem cells, which may arise from the uncoupling of proliferation and differentiation processes, remain a critical focus of research aimed at preventing recurrence and eradicating residual disease [84]. Despite significant progress in detection and treatment, cancer continues to pose a major global health burden, necessitating further research to develop more effective therapies and deepen our understanding of the underlying mechanisms driving cancer progression [85].

## In vivo applications of nanotechnology

Nanotechnology, defined as the manipulation of matter at the atomic and molecular scale (1–100 nanometers), is increasingly recognized for its potential to transform medicine [86]. Its in vivo applications offer groundbreaking opportunities for diagnosing, treating, and monitoring diseases with unprecedented precision at the cellular and molecular levels, paving the way for innovative therapeutic strategies and personalized medicine [87].

## Drug delivery systems

### Targeted Drug Delivery

Nanoparticles (NPs) are engineered to modify the pharmacokinetics and enhance the activity of drugs by serving as carriers for targeted delivery to specific cells or tissues. This precision targeting minimizes systemic side effects while maximizing the therapeutic index of the drug. A prime example in cancer treatment is the use of nanoparticles functionalized with ligands, such as folic acid, which bind to overexpressed receptors on tumor cells, such as folate receptors in folate receptor-positive tumors. This approach ensures that the drug is delivered directly to cancer cells, improving efficacy and reducing harm to healthy tissues [88]. This direct manipulation of molecules is designed to precisely deliver therapeutics to the tumor microenvironment, enhancing their efficacy while minimizing off-target effects. By concentrating treatment at the site of the tumor, this approach aims to improve therapeutic outcomes and reduce damage to healthy tissues, offering a more targeted and effective strategy for cancer therapy.

### Types of nanocarriers

Nanotechnology leverages small molecular structures and particles to revolutionize drug delivery and development. Among the most widely utilized nanocarriers in cancer treatment are liposomes, polymeric nanoparticles, dendritic macromolecules, quantum dots, and carbon nanotubes. These advanced systems enable precise drug delivery, enhanced therapeutic efficacy, and reduced side effects, making them indispensable tools in modern oncology [89].

- *Liposomes*: Liposomes are spherical vesicles consisting of a lipid bilayer, capable of encapsulating both hydrophilic and lipophilic drugs. Compared to conventional liposomes, those modified with polyethylene glycol (PEG) conjugates—through a process known as PEGylation—exhibit significantly prolonged blood circulation times. This enhancement improves drug stability and bioavailability, making PEGylated liposomes a highly effective delivery system for therapeutic agents [90]. The enhanced permeability and retention (EPR) effect serves as a critical mechanism for targeted drug delivery to tumors, leveraging the abnormal vasculature of tumor tissues that allows therapeutic agents to extravasate through gaps in the blood vessel walls. Notably, smaller liposomes, particularly those around 100 nm in size, demonstrate superior tumor penetration and prolonged systemic circulation, enhancing their therapeutic efficacy [91]. Clinically, liposomal formulations such as liposomal adriamycin have been successfully employed in cancer therapy, showing promising outcomes in the treatment of aggressive cancers, including metastatic ovarian cancer. These advancements underscore the potential of nanotechnology-based drug delivery systems in improving cancer treatment strategies [92]. A notable example of a successful application is Doxil ®, a liposomal formulation of doxorubicin ®, which demonstrates reduced cardiotoxicity compared to the free drug, highlighting the potential of nanoparticle-based systems to improve the safety and efficacy of cancer treatments [93]
- *Polymeric Nanoparticles (PNPs)*: Polymeric Nanoparticles (PNPs) represent a promising class of drug delivery systems composed of biodegradable polymers, such as poly(lactic‒glycolic acid) (PLGA), with sizes ranging from 10 to 1000 nm [94]. These nanoparticles are particularly effective in delivering hydrophobic drugs, which are often essential in cancer therapy. PNPs exhibit the ability to spontaneously self-assemble in aqueous environments and can be engineered for triggered drug release in response to specific stimuli, such as pH or temperature changes [95]. Their high stability and controlled penetration into tissues contribute to reduced toxicity to healthy, non-targeted cells, enhancing their therapeutic safety profile. However, challenges remain in achieving selective targeting of tumor cells while minimizing off-target effects and side effects [96].
- *Dendrimers*: Dendrimers are highly structured nanoparticles characterized by a spherical polymer core and symmetrically branched architectures, typically synthesized through divergent or convergent methods using various molecules such as polyacrylamide and polylysine [97]. Their unique structure allows for the attachment of multiple drug molecules to the surface, enabling controlled and sustained drug release at target sites. The tunable properties of dendrimers, including their size, surface charge, and functionalization capabilities, make them particularly effective for targeting cancer cells, which often exhibit specific characteristics such as overexpression of folate receptors [98]. These features, combined with their ability to maintain controlled drug release at tumor sites, position dendrimers as promising agents for targeted cancer therapy. Their precision in drug delivery minimizes off-target effects and enhances therapeutic efficacy, underscoring their potential in advancing cancer treatment strategies[99]
- *Carbon nanotubes*: Single-wall carbon nanotubes (SWNTs) and multi-walled carbon nanotubes (MWCNTs) are distinguished by their internal structures and outer diameters. SWNTs consist of a single cylindrical graphene layer, while MWCNTs are composed of multiple concentric graphene layers nested within one another. Carbon nanotubes (CNTs) exhibit exceptional physical and chemical properties, including high surface area, mechanical strength, and superior electrical and thermal conductivity, making them highly suitable for large-scale biomedical applications [100]. Notably, CNTs can absorb near-infrared (NIR) light, generating thermal energy that can be utilized to selectively target and destroy cancer cells. Additionally, their ability to penetrate biofilms noninvasively and efficiently deliver drugs into living cells highlights their potential in drug delivery systems. For instance, CNTs have been successfully employed to deliver anticancer drugs such as paclitaxel, demonstrating efficacy in both in vitro and in vivo settings. These properties underscore the versatility of CNTs as promising tools in cancer therapy and biomedical research [101].
- *Quantum dots*: Quantum dots (QDs) are nanoscale semiconductor crystals renowned for their unique photophysical properties, including excellent light stability, high quantum yield, and superior brightness. These characteristics make QDs highly advantageous for biomedical applications, particularly in cancer diagnosis and therapy. Techniques such as QD conjugates and quantum dot immunostaining enhance multiplexing capabilities and offer cost-effective solutions for detecting tumor biomarkers. For instance, QD-based immunostaining has demonstrated remarkable sensitivity in identifying low-expression protein biomarkers, such as HER2, enabling more accurate cancer diagnostics. Additionally, QDs can be engineered for targeted drug delivery, potentially reducing the side effects associated with conventional chemotherapy. Surface modifications further enhance their specificity for cancer therapy and imaging applications. Compared to traditional fluorescent dyes, QDs emit brighter and more stable signals, making them ideal for high-sensitivity assays and multicolor fluorescence imaging. Examples include the use of QD probes for in vitro detection of tumor markers like CA125 and immunohistochemistry (IHC) applications, underscoring their potential to revolutionize cancer diagnostics and therapeutic strategies [102].

### Benefits of nanoparticle-based drug delivery

The application of nanoparticles significantly enhances the solubility of poorly water-soluble drugs, thereby improving their bioavailability [103]. By enabling targeted drug delivery to specific sites of action, nanoparticles reduce the exposure of healthy tissues to toxic agents, minimizing adverse effects [104]. Furthermore, nanoparticles can be engineered to achieve controlled drug release, either in a sustained manner or in response to specific stimuli such as pH or temperature changes. This capability allows for prolonged therapeutic effects and reduces the frequency of dosing, enhancing patient compliance and treatment efficacy. These attributes underscore the potential of nanoparticle-based drug delivery systems to optimize therapeutic outcomes while mitigating toxicity and improving patient care [105].

## Diagnostic applications

### Imaging technologies

Nanotechnology has significantly advanced the field of medical imaging, providing high-resolution images that are critical for early diagnosis and precise treatment planning. Superparamagnetic iron oxide nanoparticles (SPIONs) have emerged as highly effective MRI contrast agents, enhancing the differentiation between tissues and improving the early detection of tumors [106]. Similarly, gold nanoparticles, with their high X-ray attenuation properties, are being utilized in computed tomography (CT) imaging to achieve superior visualization and resolution of blood vessels and tumors [107]. Additionally, radiolabeled nanoparticles are proving invaluable in positron emission tomography (PET) scans, offering unprecedented insights into molecular and cellular processes within the body. These innovations underscore the transformative potential of nanotechnology in enhancing the accuracy, sensitivity, and utility of medical imaging techniques, ultimately leading to better patient outcomes [108].

### Biosensors and biomarkers

Nanotechnology, in conjunction with microelectromechanical systems (MEMS), has enabled groundbreaking advancements in single-molecule imaging and ultrasensitive biosensing [109]. These biosensors are capable of detecting disease biomarkers, such as proteins, DNA, or RNA, at exceptionally low concentrations (as low as pg/mL), facilitating the early diagnosis of conditions like cancer and cardiovascular diseases [110]. Furthermore, nanoparticles can be engineered for real-time, in vivo monitoring of disease progression and treatment efficacy. For instance, fluorescent quantum dots (QDs) can be employed to track the spatiotemporal distribution and accumulation of therapeutic agents within the body. This capability provides critical insights into drug behavior and enhances the ability to predict treatment outcomes more reliably [111]. These innovations highlight the transformative potential of nanotechnology in improving diagnostic accuracy, enabling personalized medicine, and optimizing therapeutic strategies.

Biosensor analytical techniques are broadly classified into three categories: direct assays, sandwich assays, and competitive assays [112]. Among these, the sandwich assay is a widely used paradigm in which the target antigen is captured between two antibodies. In this approach, capture antibodies are first immobilized on the transducer surface, where they bind to the target antigen. Subsequently, detection antibodies, which are labeled with signal-generating indicators such as enzymes, fluorescent dyes, or redox compounds, bind to the antigen, completing the "sandwich" structure. This design not only enhances specificity but also allows for signal amplification, enabling highly sensitive detection of target molecules [113]. The versatility and precision of sandwich assays make them a cornerstone in biosensing applications, particularly for the detection of biomarkers in diagnostics and research [114]. Gold nanoparticles (AuNPs) are particularly favored in biosensing and biomedical applications due to their exceptional properties, including high electrical conductivity, ease of synthesis, excellent biocompatibility, and remarkable stability under oxidizing conditions [115]. These characteristics make AuNPs ideal for use in various diagnostic and therapeutic platforms. For instance, their high conductivity enhances signal transduction in electrochemical biosensors, while their biocompatibility ensures minimal toxicity in biological systems. Additionally, their stability allows for reliable performance in diverse environments, further solidifying their role as a versatile and effective tool in nanotechnology-based applications [116].

One of the key features that make gold nanoparticles highly effective for biosensing applications is localized surface plasmon resonance (LSPR), an optical phenomenon observed in noble metal nanostructures such as gold and silver [117, 118]. LSPR arises due to the collective oscillation of conduction electrons in response to light, resulting in strong absorption and scattering peaks in the visible spectrum [119, 120]. This property is particularly advantageous for nanobiosensors, as it enables highly sensitive detection of target molecules. For instance, a sandwich immunoassay utilizing second antibody–second gold nanoparticle (2nd Ab–2nd AuNP) conjugates can significantly enhance the detection limit of fiber-optic LSPR (FO LSPR) biosensors [121]. In this approach, the sequential binding of 2nd Ab–2nd AuNP conjugates to antigens amplifies the LSPR signal on the fiber-optic surface, leading to improved sensitivity and signal output [122]. In a commercial avian influenza detection kit, gold nanoparticles (AuNPs) were utilized as carriers for signaling anti-chicken antibody peroxidase. Compared to systems employing anti-chicken antibody peroxidase alone, the incorporation of AuNPs significantly enhanced absorbance and reduced assay time. This improvement is attributed to the unique ability of AuNPs to carry multiple enzyme molecules, which amplifies the enzymatic reaction with the substrate. As a result, the optical signal is substantially enhanced while maintaining low background noise, leading to higher sensitivity and faster detection. This example highlights the practical advantages of AuNPs in biosensing, demonstrating their capacity to improve assay performance through signal amplification and efficiency [123, 124].

### Regenerative medicine

Scaffolds designed to mimic the native extracellular matrix (ECM) are increasingly being developed using nanomaterials to support structural growth and facilitate cell and organ regeneration. These scaffolds can be functionalized with growth factors, peptides, or other bioactive molecules to enhance critical cellular processes such as adhesion, proliferation, and differentiation. By closely replicating the natural ECM environment, these nanomaterial-based scaffolds provide a conducive platform for tissue engineering and regenerative medicine. The incorporation of bioactive elements further tailors the scaffolds to specific therapeutic needs, improving their effectiveness in promoting tissue repair and regeneration. This approach underscores the potential of nanotechnology to advance regenerative therapies and address complex challenges in tissue engineering [125]. For example, collagen or chitosan have since been utilized for wound healing in bone, cartilage, and skin tissues [126]. Nanotechnology plays a pivotal role in enhancing stem cell-based therapies by improving the retention and targeted delivery of stem cells to specific sites, such as atherosclerotic lesions or gene target sites [127]. Magnetic nanoparticles, for instance, enable the precise guidance of stem cells to desired locations using external magnetic fields, increasing the efficiency of cell delivery. Additionally, nanoparticles serve as effective vehicles for delivering growth factors or genes that direct stem cell differentiation into specific lineages, thereby enhancing their therapeutic potential. These advancements not only improve the precision and efficacy of stem cell treatments but also contribute to the development of more effective regenerative therapies for a wide range of medical conditions [128].

### Immunotherapy

Nanotechnology has significantly advanced the field of immunotherapy, particularly in vaccine development and the modulation of immune responses. A key challenge in vaccine development is ensuring efficient delivery of antigens to immune cells. Nanoparticles, such as aluminum-based adjuvants, address this issue by facilitating the direct delivery of antigens to antigen-presenting cells (APCs), such as dendritic cells and macrophages. This targeted delivery enhances the specificity and intensity of the immune response, leading to more effective vaccination outcomes. By improving antigen uptake and presentation, nanoparticle-based systems not only boost the efficacy of vaccines but also enable the development of novel immunotherapies for a wide range of diseases, including cancer and infectious diseases. This highlights the transformative potential of nanotechnology in revolutionizing immunotherapy and vaccine design [129]. Nanotechnology enables precise modulation of the immune response by facilitating the targeted and efficient delivery of immunomodulatory agents, such as cytokines, antibodies, or small molecules, directly to immune cells. This selective delivery enhances the immune system's ability to combat diseases, including cancer and autoimmune disorders, by optimizing therapeutic efficacy while minimizing off-target effects and systemic toxicity [130]. For example, nanoparticles functionalized with anti-PD-1 antibodies offer a promising strategy in cancer immunotherapy by blocking the interaction between the programmed cell death protein 1 (PD-1) receptor on T cells and its ligands on tumor cells. This blockade prevents tumor cells from evading immune recognition, thereby restoring the ability of T cells to recognize and attack cancer cells. By delivering anti-PD-1 antibodies directly to the tumor microenvironment, nanoparticle-based systems enhance the precision and efficacy of immune checkpoint inhibition while minimizing systemic side effects. This targeted approach not only improves the therapeutic outcomes of immune checkpoint therapy but also represents a significant advancement in the fight against cancer by leveraging the immune system's natural ability to combat malignancies [131].

## Mechanisms by which nanotechnology can treat cancer

Nanotechnology has revolutionized cancer therapy by leveraging the enhanced permeability and retention (EPR) effect and active targeting mechanisms, such as aptamers, to achieve precise drug delivery to tumor sites. This approach minimizes off-target effects and reduces undesirable side effects on healthy tissues [99]. Additionally, nanotechnology facilitates the delivery of genetic materials for gene therapy and RNA interference (RNAi), enabling the silencing of oncogenes and offering new avenues for cancer treatment [132].

In the realm of immunotherapy, nanotechnology enhances immune system effectiveness and supports the development of personalized cancer vaccines, tailoring treatments to individual patients [133]. It also plays a critical role in reshaping the tumor microenvironment, making it less conducive to cancer progression. Furthermore, nanotechnology advances early cancer diagnosis and theranostics—combining targeted imaging of nucleic acids or proteins with therapeutic monitoring—to improve treatment outcomes and enable real-time tracking of therapeutic responses [134]. These innovations underscore the transformative potential of nanotechnology in improving cancer therapy, diagnosis, and personalized medicine.

### Targeted drug delivery to tumors

Nanotechnology has revolutionized tumor-specific drug delivery by harnessing mechanisms such as the enhanced permeability and retention (EPR) effect, active targeting strategies, and regulated release processes to enhance drug control and efficacy. The EPR effect, a hallmark of tumor biology, allows nanoparticles to accumulate preferentially in tumor tissues due to the leaky vasculature and impaired lymphatic drainage of tumors. This results in prolonged retention and higher local concentrations of therapeutic agents, which is particularly beneficial for chemotherapeutic drug delivery, as it reduces systemic toxicity and improves treatment outcomes [135].

Beyond passive targeting, nanoparticles can be actively targeted to cancer cells through surface functionalization with ligands such as antibodies or peptides. These ligands bind specifically to receptors overexpressed on tumor cells, enabling precise drug delivery. For example, HER2 ® -targeted nanoparticles conjugated with trastuzumab ® are used to treat HER2-positive breast cancer by delivering drugs directly to cells overexpressing the HER2 receptor [136]. Similarly, folate ® receptor-targeted nanoparticles exploit the high expression of folate receptors on certain tumor types, enabling direct targeting of cancer cells and reducing off-target effects. This approach is particularly effective in delivering chemotherapeutic agents to circulating tumor cells (CTCs) and minimizing toxic side effects [137].

Additionally, nanotechnology enables the development of stimuli-responsive drug release systems, where therapeutic payloads are released in response to specific conditions within the tumor microenvironment, such as acidic pH, elevated temperature, or enzymatic activity [138]. These smart delivery systems ensure that drugs are activated precisely at the tumor site, minimizing exposure to healthy tissues and reducing off-target effects [139]. By leveraging the unique characteristics of the tumor microenvironment, such as its lower pH or higher enzymatic activity, nanotechnology allows for controlled and targeted drug release, enhancing the precision and efficacy of cancer therapy . These advancements underscore the transformative potential of nanotechnology in revolutionizing cancer treatment. By improving the specificity, efficacy, and safety of therapeutic interventions, nanotechnology paves the way for more personalized and effective cancer therapies, ultimately leading to better patient outcomes [140].

### Nanoparticles in chemotherapy

Nanotechnology has emerged as a powerful tool to enhance the efficacy of chemotherapy while mitigating its adverse side effects and addressing drug resistance. Traditional chemotherapy drugs often cause severe side effects due to their nonspecific distribution throughout the body. Nanoparticles (NPs) address this challenge by enabling selective drug delivery to tumor sites, thereby minimizing exposure to healthy tissues and reducing systemic toxicity [141, 142]. For instance, encapsulating doxorubicin within liposomes (e.g., Doxil ®) significantly reduces cardiac toxicity while maintaining its antitumor efficacy [143, 144]. Similarly, paclitaxel-loaded nanoparticles (e.g., Abraxane ®), which use albumin-bound nanoparticles, enhance drug localization at tumor sites and reduce peripheral side effects compared to traditional paclitaxel formulations [145].

Moreover, nanotechnology provides a promising solution to chemotherapeutic drug resistance, a significant challenge in cancer treatment. Drug resistance often occurs due to mechanisms such as the overexpression of efflux pumps like P-glycoprotein, which actively expel chemotherapeutic agents from cancer cells, reducing their effectiveness [146]. Nanoparticles (NPs) address this issue by enabling direct delivery of drugs into the cytoplasm or nucleus of cancer cells, bypassing these efflux pumps and ensuring higher intracellular drug concentrations. Furthermore, NPs can codeliver efflux pump inhibitors alongside chemotherapeutic agents, effectively blocking the activity of resistance mechanisms and enhancing the overall efficacy of the treatment [147]. This dual approach not only overcomes drug resistance but also improves the therapeutic outcomes of chemotherapy. By leveraging the unique capabilities of nanotechnology, researchers are developing more effective strategies to combat drug-resistant cancers, offering hope for improved patient outcomes and more durable responses to treatment

### Gene therapy

Nanoparticles have emerged as highly efficient gene carriers, playing a pivotal role in advancing gene therapy. They protect genetic material, such as DNA or RNA, from degradation by extracellular nucleases while enhancing cellular uptake and targeted delivery [148, 149]. For example, lipid nanoparticles have shown great promise in delivering siRNAs or mRNAs, enabling the silencing of oncogenes or restoring the function of tumor suppressor genes in cancer therapy [150]. Moreover, nanotechnology is driving innovations in the delivery of CRISPR/Cas9 systems, which allow for precise gene editing. This technology holds immense potential for correcting oncogenic mutations in tumor cells [151]. For instance, studies have demonstrated that the overexpression of long non-coding RNA SNHG9 can enhance the pharmacological effects of gene silencing using the Si-CRISPR/Cas system in vitro [152]. By encapsulating CRISPR/Cas9 components into nanoparticles, researchers have improved the specificity and efficiency of gene editing while minimizing off-target effects and reducing unintended nuclease-induced alterations during cellular DNA repair processes [153]. These advancements underscore the transformative potential of nanotechnology in gene therapy, offering precise and effective tools for targeting genetic abnormalities in cancer and other diseases. By enhancing the delivery and accuracy of gene-editing technologies, nanoparticles are paving the way for groundbreaking therapeutic strategies.

### Photothermal and photodynamic therapies

Nanoparticles serve as a versatile platform for enhancing cancer treatment through light-based therapies, primarily photothermal therapy (PTT) and photodynamic therapy (PDT) [154]. In PTT, nanoparticles such as gold particles absorb near-infrared (NIR) light and convert it into heat, selectively destroying cancer cells while sparing surrounding healthy tissues [155]. In contrast, PDT relies on nanoparticles to deliver photosensitizing agents to tumor tissues, where they generate reactive oxygen species (ROS) upon exposure to specific wavelengths of light, leading to cancer cell death [156]. Gold nanoparticles and certain organic nanoparticles are particularly effective in these applications. For instance, porphyrin and phthalocyanine photosensitizers encapsulated in silica nanoparticles enhance PDT efficacy by improving ROS generation and tumor targeting [157]. These light-activated therapies represent a promising approach for precise and minimally invasive cancer treatment.

### Immune modulation and cancer vaccines

Nanoparticles are revolutionizing cancer therapy by enabling innovative approaches such as nanoparticle-based cancer vaccines and modulation of the tumor microenvironment (TME). These advancements hold significant promise for enhancing adjuvant therapy and improving cancer treatment outcomes. For instance, dendritic cell-targeting nanoparticle-based cancer vaccines can deliver tumor-associated antigens, priming the immune system to recognize and destroy tumors. Polymeric nanoparticles have been successfully employed to deliver both antigens and adjuvants, effectively stimulating robust antitumor immune responses [158].

Additionally, nanoparticles play a critical role in reshaping TME, which is often an immune-suppressive niche that fosters tumor growth and progression. By delivering agents that deplete immunosuppressive cells, such as regulatory T cells (Tregs) or myeloid-derived suppressor cells (MDSCs), nanoparticles can reverse immune suppression [159]. This restoration of immune competence enhances antitumor responses and improves the efficacy of immune checkpoint inhibitors. These strategies highlight the transformative potential of nanotechnology in cancer immunotherapy, offering new ways to harness the immune system for more effective and targeted cancer treatment [134]. By combining vaccine development with TME modulation, nanoparticles are paving the way for innovative and personalized therapeutic approaches.

### Theranostic nanoplatforms for various cancer therapy approaches

The term "theranostics" was first used to describe a method that combines treatments and diagnostics. The procedure employs a variety of methods to produce molecular images, a thorough diagnosis, and a customized treatment plan [160].

A theranostic platform has the potential to be transformative by encapsulating a tiny gold cluster and the anticancer medication doxorubicin. We present a multifunctional theranostic nanostructure that can enhance cancer diagnosis and treatment through improved chemotherapy, radiotherapy, and existing X-ray imaging technologies. Gold clusters have been utilized to treat malignant cells via in vitro hyperthermia and provide good heating when exposed to radiofrequency electric fields (RF-EFs). Liposomal doxorubicin has been synthesized and investigated as a theranostic drug with high potential for various cancer therapy modalities [91].

## Disadvantages of nanoparticles in cancer therapy

**Fig. 9:**
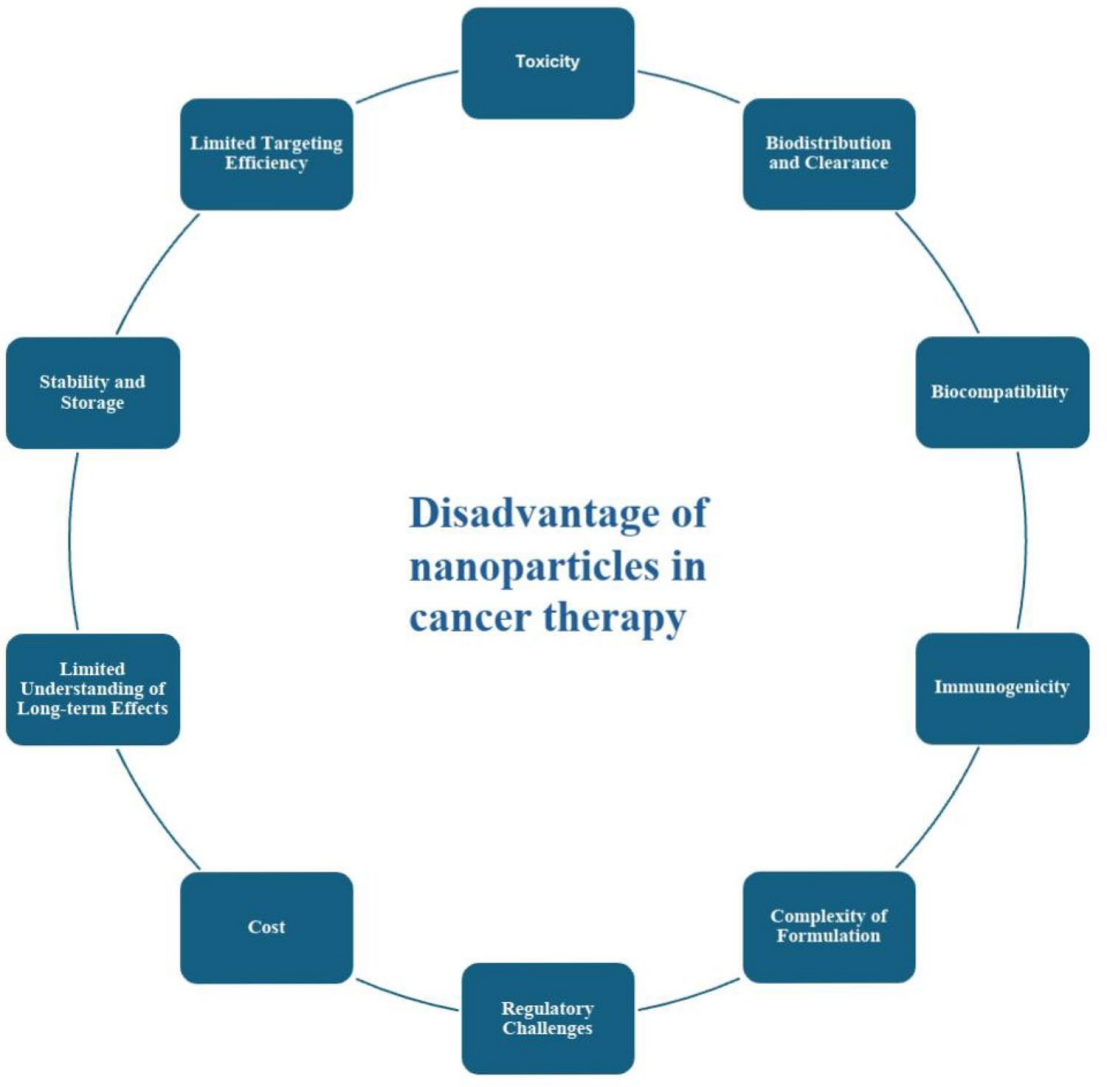
Schematic diagram showing the disadvantages of using nanoparticles in cancer treatment

Nanoparticles have shown great promise in cancer therapy, but they also have several disadvantages. As shown in Fig. 9 some of the key drawbacks are as follows:

1. *Toxicity*: Certain nanoparticles, particularly metal-based ones, can induce cytotoxicity in healthy cells, leading to oxidative stress, inflammation, and damage to normal tissues and organs [161].

2. *Biodistribution and clearance*: Nanoparticles may exhibit unpredictable biodistribution, accumulating in non-target organs and causing potential toxicity. Smaller nanoparticles are prone to aggregation, which can hinder their ability to reach tumor sites effectively. Additionally, variability in clearance rates may lead to prolonged exposure and associated toxicity [162].

3. *Biocompatibility*: Not all nanoparticles are biocompatible, and some may trigger adverse immune or inflammatory responses. Ensuring biocompatibility is critical for their successful therapeutic application [163].

4. *Immunogenicity*: Nanoparticles can be recognized as foreign bodies by the immune system, leading to rapid clearance and reduced therapeutic efficacy. This immunogenicity poses a significant challenge for their use in therapy [164].

5. *Complexity of formulation*: Designing nanoparticles that balance effective drug delivery with minimal toxicity is highly complex. Factors such as surface chemistry, size, and shape must be meticulously optimized, complicating the manufacturing process [88].

6. *Regulatory challenges*: The unique properties of nanoparticles necessitate new safety assessments and regulatory frameworks, which can delay their clinical translation and approval [165].

7. *Cost*: The production, purification, and quality control of nanoparticles can be expensive, potentially limiting their accessibility and widespread adoption in clinical settings [166].

8. *Limited understanding of long-term effects*: There is insufficient knowledge about the long-term effects of nanoparticle exposure in humans, including chronic toxicity and potential carcinogenicity [167]

9. *Stability and Storage*: Nanoparticles are often sensitive to environmental conditions, which can affect their stability and therapeutic efficacy. Proper storage and handling are essential to maintain their functionality [168].

10. *Limited Targeting Efficiency (Limited Penetration)*: Despite advances in targeted delivery, achieving precise targeting remains challenging due to tumor heterogeneity and variations in the tumor microenvironment. Additionally, the dense extracellular matrix of solid tumors can impede nanoparticle penetration, reducing their effectiveness in delivering therapeutic agents [169].

## Microbial anticancer therapy

### Definition

Microbial anticancer therapy involves the utilization of microorganisms, including bacteria or their components, for the prevention, treatment, or management of cancer [170]. This innovative approach capitalizes on the unique ability of specific microbes to stimulate the immune system, promote tumor regression, or augment the effectiveness of conventional cancer therapies [171]. Historically, this concept was exemplified by Coley’s toxins, derived from *Streptococcus pyogenes* and *Bacillus prodigiosus*, which were used to trigger an immune response against tumors [172]. In contemporary research, the focus has shifted to understanding the role of the gut microbiota in shaping cancer therapy outcomes, particularly in immunotherapy and chemotherapy, by modulating immune responses and enhancing therapeutic efficacy [173].

### Bacteria-based cancer treatment

Bacteria-based cancer treatment is a therapeutic approach that employs natural or genetically engineered bacteria to prevent, target, or treat cancer [174]. This strategy leverages the unique properties of bacteria, such as their ability to selectively accumulate in tumor tissues, stimulate immune responses, and produce anticancer compounds, to enhance the effectiveness of cancer therapies. The approach may involve the use of live bacteria, bacterial components (e.g., toxins or enzymes), or genetically modified bacteria designed to express therapeutic proteins or deliver drugs directly to cancer cells [175]. This innovative method holds significant promise for improving treatment specificity, reducing side effects associated with conventional therapies, and ultimately enhancing patient outcomes in cancer care [176].

### Some types of bacteria that are utilized in cancer

- *Salmonella* Typhimurium: *S.* Typhimurium is recognized for its unique capability to selectively accumulate in hypoxic tumor microenvironments, where it enhances both innate and adaptive immune responses, thereby promoting antitumor activity [177].
- *Mycobacterium bovis*: *M. bovis*, particularly its attenuated strain Bacillus Calmette-Guérin (BCG), is utilized in the treatment of superficial noninvasive bladder cancer. BCG activates immune responses that induce tumor cell death, making it a cornerstone of immunotherapy for this cancer type [178].
- *Listeria monocytogenes*: *L. monocytogenes*, a gram-positive bacterium, is highly effective in delivering cancer cell antigens and eliciting robust immune responses. Its ability to escape phagosomes within macrophages enhances its potential as a vehicle for cancer immunotherapy. [179].
- *Escherichia coli*: Certain strains of *E. coli*, especially probiotic lactic acid bacteria, are employed in the treatment of various cancers, such as colon and breast cancer, due to their therapeutic potential and ability to modulate the tumor microenvironment [180].
- *Pseudomonas aeruginosa*: *P. aeruginosa* has demonstrated promising antitumor activity by inhibiting tumor growth in specific mouse models, highlighting its potential as a novel therapeutic agent in cancer research [181].

### Viruses

Oncolytic viruses, whether genetically engineered or naturally occurring, are designed to selectively replicate within and destroy tumor cells while sparing normal cells, offering a targeted and minimally invasive approach to cancer therapy [182]. Examples of these viruses are detailed in Table 3.

**Table 3:** Some virus vaccine specifications from the advanced clinical development stage

Name of the virus	type	Indication	Mechanism	References
Talimogene Laherparepvec (T-VEC)	Genetically modified herpes simplex virus (HSV)	Advanced melanoma	Infects and lyses tumor cells while expressing the human granulocyte–macrophage colony-stimulating factor (GM-CSF) to enhance immune responses by attracting immune cells to the tumor site	[183]
H101	Genetically modified adenovirus	Head and neck cancer	Infects cancer cells, causing lysis and releasing viral particles to infect neighboring tumor cells, stimulating an immune response against the tumor	[184]
Reovirus	Naturally occurring double-stranded RNA virus	Various solid tumors, including breast and pancreatic cancer	Selectively infects cancer cells with activated Ras pathways, exploiting dysregulated signaling to replicate and induce cell lysis, sparing normal cells with intact Ras signaling	[185]
Newcastle Disease Virus (NDV)	Avian paramyxovirus	Various cancers, including breast and lung cancer	Induces tumor cell death via apoptosis and necrosis, exploits altered signaling pathways in cancer cells, and stimulates an immune response to enhance antitumor effects	[186]
Seneca Valley Virus (SVV)	Naturally occurring RNA virus	Neuroendocrine tumors and other cancers	Targets tumor cells via unique surface receptors, replicates within cancer cells, and causes lysis, releasing viral particles to infect neighboring cells and amplify tumor destruction	[187]
Vaccinia Virus	Poxvirus	Melanoma and other solid tumors	Induces cell death via apoptosis and necrosis, stimulates antitumor immune responses, and can express foreign genes (e.g., cytokines) to further enhance immune activity	[188]
Measles Virus	Single-stranded RNA virus	Multiple myeloma and other cancers	Enters tumor cells via natural receptors, replicates, and induces lysis, with the immune response against infected cells enhancing the destruction of neighboring cancer cells	[189]
Maraba Virus	RNA virus	Various cancers	Induces tumor cell death via apoptosis and necrosis, stimulates immune responses, and replicates within tumor cells to release new viral particles that infect neighboring cancer cells	[190]
Poliovirus	Enterovirus	Glioblastoma	Enters tumor cells via specific receptors, replicates, and induces lysis, with the immune response against infected cells enhancing the destruction of neighboring cancer cells	[191]
ECHO-7 (RIGVIR)	Enterovirus	Skin melanoma	Enters tumor cells via specific receptors, replicates, and induces apoptosis, while stimulating an immune response that targets the tumor	[192]

*Fungi*: Fungi, particularly certain species of mushrooms, are recognized for their ability to produce bioactive compounds with anticancer properties [193]. These compounds exhibit immune-boosting effects and demonstrate potential antitumor activity, making them valuable candidates for further exploration in cancer therapy and prevention [194].

*Archaea*: Archaea, though less extensively studied than bacteria and fungi, possess unique metabolic properties that may offer potential benefits in cancer therapy. Their distinct biochemical pathways and adaptations to extreme environments could provide novel insights and therapeutic strategies for targeting cancer metabolism and progression [195].

*Probiotics*: Probiotics and prebiotics, which consist of beneficial microbes and substances that support the growth of healthy gut bacteria, have shown potential to enhance the efficacy of cancer treatments. By modulating the gut microbiota and immune responses, they may improve therapeutic outcomes and contribute to a more robust antitumor immune environment [196].

### Plants as anticancer agents

The exploration of plant-derived compounds for their anticancer properties underscores the significant potential of natural substances in both cancer prevention and treatment. These plants are rich in bioactive compounds capable of inhibiting tumor growth, inducing apoptosis, and modulating immune responses. A deeper understanding of their mechanisms of action and the development of effective plant-based therapies are critical for advancing cancer treatment strategies and harnessing the full therapeutic potential of natural products [197]. Plant-derived compounds are utilized as chemical reducing agents in the synthesis of metallic nanoparticles with anticancer activity, leveraging their natural bioactive properties to facilitate eco-friendly and sustainable nanoparticle production [198]. In this regard, the flavonoid apigenin (Api), a green sensitizer, stabilizes nanoparticles and promotes the destruction of cancer cells by inhibiting DNA damage repair, highlighting its potential as a key component in nanoparticle-based anticancer therapies [199]. Additionally, Api induces apoptosis, suppresses cell proliferation, and arrests cell cycle progression in the G0/G1 phase, particularly when combined with radiation, enhancing its anticancer efficacy [200].

Curcumin® a naturally occurring phenolic compound derived from *Curcuma longa*, has been extensively studied for its diverse biological applications. Research has demonstrated its ability to enhance cancer treatment, inhibit metastasis, and protect healthy cells from radiation-induced damage [201]. Curcumin ® also exhibits anti-aging properties in humans, animals, and experimental models [202]. Furthermore, it serves as an effective reducing and capping agent in the synthesis of gold nanoparticles, with curcumin-coated gold nanoparticles (Cur-AuNPs) being widely explored for various biomedical applications [203]. A comprehensive review of Cur-AuNP formulations highlights their potential in the prevention and treatment of numerous diseases [204].

In addition, selenium nanoparticles (SeNPs) have shown significant antibacterial and antibiofilm properties [205]. Recent studies suggest that SeNPs may enhance the efficacy of photodynamic therapy (PDT), with current research evaluating their activity in combination with PDT against planktonic microbial communities [206].

Bismuth ® oxide nanoparticles, synthesized using sorbitol under optimized conditions, have been tested for their radiation-shielding properties using X-ray dosimeters. While lead aprons are currently used for radiation protection, their drawbacks—such as weight, discomfort, high cost, and environmental hazards—highlight the need for alternative solutions [207]. Developing lightweight, non-toxic, and eco-friendly radiation shields remains a critical challenge in advancing radiation protection technologies [208].

Various extraction techniques are employed to isolate bioactive compounds from medicinal plants [209], with the goal of maximizing the yield of target compounds while minimizing the presence of unwanted components. These methods are essential for obtaining high-quality extracts for therapeutic and research applications.

## The key extraction techniques mentioned include the following

1. *Maceration*: This is a straightforward and widely used extraction technique in which plant materials are soaked in a suitable solvent at room temperature for a specified period. During this process, the solvent penetrates the plant tissues, allowing the soluble compounds to diffuse out. Maceration is particularly effective for extracting heat-sensitive bioactive compounds that may degrade under high temperatures. It is a cost-effective and low-energy method, making it a popular choice for preliminary extraction of phytochemicals from plant sources. However, the efficiency of maceration depends on factors such as the type of solvent, particle size of the plant material, and duration of soaking [210].

2. *Percolation*: This method involves the continuous flow of a solvent through a column packed with finely ground plant material, facilitating the efficient extraction of bioactive compounds. Percolation is particularly effective for extracting compounds from solid plant matrices, as the solvent systematically penetrates the material, ensuring thorough extraction. This technique offers advantages such as higher extraction efficiency, reduced solvent consumption, and shorter processing times compared to other methods. Its adaptability and scalability make it a preferred choice for the extraction of phytochemicals in both laboratory and industrial settings [211].

3. *Supercritical fluid extraction (SFE)*: This advanced and highly efficient extraction technique utilizes supercritical fluids, most commonly carbon dioxide (CO₂), as solvents to isolate bioactive compounds from plant materials. SFE operates under high pressure and temperature conditions, where the solvent exhibits properties intermediate between a liquid and a gas, enabling superior penetration and selective extraction of target compounds. A key advantage of SFE is its ability to extract thermally sensitive compounds without causing degradation, preserving their bioactivity and integrity. Additionally, SFE is environmentally friendly, as it avoids the use of toxic organic solvents and allows for easy solvent recovery. These features make SFE a preferred method for extracting high-value compounds in pharmaceutical, food, and cosmetic industries [212].

4. *Ultrasonic Extraction*: Uses ultrasonic waves to agitate solvent-plant mixtures, enhancing extraction via cavitation. Increases solvent-plant contact, enabling rapid release of bioactive compounds. Offers shorter extraction times, lower solvent use, and is ideal for rigid matrices and heat-sensitive compounds. Valued for its efficiency, simplicity, and scalability in lab and industrial settings [213].

5. *Soxhlet Extraction:* Uses a Soxhlet apparatus for continuous cycling of solvent through plant material, ensuring thorough extraction. Ideal for lipophilic compounds and valued for high efficiency and reproducibility. Requires longer time and more solvent but remains a standard method for bioactive compound extraction [214].

## Challenges and future prospects of the use of nanoparticles as novel techniques for cancer therapy

Nanoparticles have emerged as a promising tool in cancer diagnostics and treatment due to their advantages, including precise targeting, low toxicity, and high biocompatibility [215]. They address significant limitations of conventional cancer therapies, such as poor selectivity and multidrug resistance [216]. A wide variety of nanoparticles have been developed, utilizing the unique properties of tumors and their microenvironment to enable targeted drug delivery [217]. Recent advancements in nanoparticle technology have effectively addressed challenges such as prolonged circulation, enhanced tumor targeting, controlled release mechanisms, and precise subcellular localization, significantly improving their therapeutic potential [218].

Nanoparticles have shown significant potential in enhancing the efficacy of chemotherapy, combination therapies, and single-agent treatments [219]. However, the majority of research remains confined to in vitro and in vivo studies, with limited clinical applications to date. While the translation of nanoparticle-based cancer therapeutics into clinical practice presents considerable challenges, these advancements hold immense promise for improving oncological outcomes and revolutionizing cancer treatment paradigms [220].

One of the primary concerns surrounding nanoparticles is their potential toxicity. Metal-based nanoparticles, such as those composed of silver or gold, may accumulate in the body, leading to unforeseen adverse effects and long-term toxicity [221]. Consequently, there is a growing emphasis on developing biodegradable and biocompatible nanoparticles. However, the behavior of these nanoparticles within the human body remains poorly understood [222]. Future research aims to design nanoparticles that are either easily excreted or fully biodegradable. Additionally, the development of 'smart' nanoparticles, which release their therapeutic payload only in response to specific stimuli such as temperature or pH, holds promise for minimizing toxicity by ensuring they remain inactive in healthy tissues [223].

Tumor heterogeneity, characterized by varying molecular and phenotypic profiles across different regions of a tumor, presents a significant challenge for nanoparticle-based therapies. This diversity can hinder the ability of nanoparticles to uniformly target all malignant cells, potentially enabling the survival and proliferation of resistant cell populations. Overcoming this challenge requires innovative strategies to ensure comprehensive and effective targeting of heterogeneous tumor environments [224]. To address tumor heterogeneity, nanoparticles can be engineered to deliver multiple therapeutic agents, enabling them to target diverse cell populations within heterogeneous tumors. Furthermore, personalized nanoparticle-based therapies, tailored to the unique genetic and molecular profile of a patient’s tumor, hold significant potential to enhance treatment efficacy and overcome the challenges posed by tumor diversity [225].

Personalized medicine utilizing nanoparticles represents one of the most promising future directions in cancer therapy. However, this approach requires comprehensive molecular and genetic profiling of each patient, which can be both costly and time-intensive. Advances in genomic technologies are gradually making these profiling methods more accessible, paving the way for the customization of nanoparticle therapies to the unique genetic characteristics of a patient’s tumor. This tailored approach has the potential to deliver more precise and effective treatments while minimizing adverse effects, ultimately improving patient outcomes [226].

While nanoparticles are capable of delivering single therapeutic agents, their true potential lies in the delivery of combination therapies [227]. However, designing nanoparticles that can simultaneously carry multiple drugs or integrate different therapeutic modalities, such as immunotherapy and chemotherapy, remains a significant technical challenge [228]. By enabling the controlled co-delivery of chemotherapy, immunotherapy, and radiation, nanoparticles could overcome drug resistance and enhance treatment efficacy through synergistic effects, representing a transformative advancement in cancer therapy [229].

### Green chemistry

The challenges of green chemistry in the synthesis of metal and metal oxide nanoparticles lie in achieving optimal yield and efficacy while minimizing waste generation and hazardous byproducts, thereby adhering to principles of safety, cost-effectiveness, and environmental sustainability. Green chemistry represents a transformative field of study, aiming to mitigate adverse environmental and societal impacts throughout the reaction process [230]. This is accomplished through the strategic selection of reactants, reaction conditions, and catalytic additives that enhance product formation while reducing ecological footprints. Biogenic synthetic materials, characterized by their enhanced properties and robust chemical reactivity, hold significant promise for advancing nanomaterial synthesis and refinement. These methods are not only non-toxic and environmentally benign but also cost-effective and user-friendly. A wide range of metal and metal oxide nanoparticles, including titanium (Ti), vanadium (V), chromium (Cr), manganese (Mn), iron (Fe), cobalt (Co), nickel (Ni), copper (Cu), zinc (Zn), platinum (Pt), gold (Au), and silver (Ag), have been successfully synthesized using eco-friendly approaches, underscoring the versatility and potential of green chemistry in nanotechnology [231].

## Conclusions

Nanotechnology has emerged as a groundbreaking approach in cancer therapy, offering innovative solutions to overcome the limitations of conventional treatments. By leveraging the unique properties of nanoparticles, such as their ability to deliver drugs with precision, reduce systemic toxicity, and enhance therapeutic efficacy, nanotechnology has the potential to revolutionize cancer diagnosis and treatment. The development of sustainable nanocomposites derived from natural sources, such as plants and microbes, further underscores the eco-friendly and biocompatible nature of these advanced therapeutic platforms. These nanocomposites not only improve drug delivery but also minimize environmental impact, aligning with the principles of green chemistry.

Despite these advancements, challenges remain, including issues related to nanoparticle toxicity, immune system clearance, and long-term biocompatibility. Addressing these challenges through continued research and innovation is critical to ensuring the safe and effective translation of nanotechnology into clinical practice. Future directions in nanotechnology-based cancer therapy will likely focus on personalized medicine, where treatments are tailored to the genetic and molecular profiles of individual patients. Additionally, the integration of nanotechnology with combination therapies, such as immunotherapy and chemotherapy, holds promise for achieving synergistic effects and overcoming drug resistance.

In conclusion, nanotechnology represents a transformative paradigm in cancer treatment, offering unprecedented precision and efficacy. By addressing current limitations and advancing research, nanotechnology has the potential to significantly improve patient outcomes and redefine the future of oncology. The continued exploration of sustainable nanocomposites, coupled with advancements in targeted drug delivery and personalized medicine, will pave the way for safer, more effective, and environmentally friendly cancer therapies.

## Data Availability

No datasets were generated or analysed during the current study.
